# Chemokines modulate the tumour microenvironment in pituitary neuroendocrine tumours

**DOI:** 10.1186/s40478-019-0830-3

**Published:** 2019-11-08

**Authors:** Pedro Marques, Sayka Barry, Eivind Carlsen, David Collier, Amy Ronaldson, Sherine Awad, Neil Dorward, Joan Grieve, Nigel Mendoza, Samiul Muquit, Ashley B. Grossman, Frances Balkwill, Márta Korbonits

**Affiliations:** 1 Centre for Endocrinology, William Harvey Research Institute, Barts and the London School of Medicine and Dentistry, Queen Mary University of London, London, UK; 2 Department of Pathology, STHF, Skien, Norway; 3 The National Hospital for Neurology and Neurosurgery, UCLH, NHS Trust, London, UK; 4 Department of Neurosurgery, Charing Cross Hospital, Imperial College, London, UK; 5 Department of Neurosurgery, Derriford Hospital, Plymouth, UK; 6 Barts Cancer Institute, Barts and the London School of Medicine and Dentistry, Queen Mary University of London, Charterhouse Square, London, UK

**Keywords:** Pituitary neuroendocrine tumour, Pituitary adenoma, Tumour microenvironment, Cytokine, Chemokine, Immune cell, Macrophages, Lymphocytes, Neutrophils, Epithelial-to-mesenchymal transition

## Abstract

Non-tumoural cells within the tumour microenvironment (TME) influence tumour proliferation, invasiveness and angiogenesis. Little is known about TME in pituitary neuroendocrine tumours (PitNETs). We aimed to characterise the role of TME in the aggressive behaviour of PitNETs, focusing on immune cells and cytokines. The cytokine secretome of 16 clinically non-functioning PitNETs (NF-PitNETs) and 8 somatotropinomas was assessed in primary culture using an immunoassay panel with 42 cytokines. This was correlated with macrophage (CD68, HLA-DR, CD163), T-lymphocyte (CD8, CD4, FOXP3), B-lymphocyte (CD20), neutrophil (neutrophil elastase) and endothelial cells (CD31) content, compared to normal pituitaries (NPs, *n* = 5). In vitro tumour–macrophage interactions were assessed by conditioned medium (CM) of GH3 (pituitary tumour) and RAW264.7 (macrophage) cell lines on morphology, migration/invasion, epithelial-to-mesenchymal transition and cytokine secretion. IL-8, CCL2, CCL3, CCL4, CXCL10, CCL22 and CXCL1 are the main PitNET-derived cytokines. PitNETs with increased macrophage and neutrophil content had higher IL-8, CCL2, CCL3, CCL4 and CXCL1 levels. CD8+ T-lymphocytes were associated to higher CCL2, CCL4 and VEGF-A levels. PitNETs had more macrophages than NPs (*p* < 0.001), with a 3-fold increased CD163:HLA-DR macrophage ratio. PitNETs contained more CD4+ T-lymphocytes (*p* = 0.005), but fewer neutrophils (*p* = 0.047) with a 2-fold decreased CD8:CD4 ratio. NF-PitNETs secreted more cytokines and had 9 times more neutrophils than somatotropinomas (*p* = 0.002). PitNETs with higher Ki-67 had more FOXP3+ T cells, as well as lower CD68:FOXP3, CD8:CD4 and CD8:FOXP3 ratios. PitNETs with “deleterious immune phenotype” (CD68^hi^CD4^hi^FOXP3^hi^CD20^hi^) had a Ki-67 ≥ 3%. CD163:HLA-DR macrophage ratio was positively correlated with microvessel density (*p* = 0.015) and area (*p* < 0.001). GH3 cell-CM increased macrophage chemotaxis, while macrophage-CM changed morphology, invasion, epithelial-to-mesenchymal transition and secreted cytokines of GH3 cells. PitNETs are characterised by increased CD163:HLA-DR macrophage and reduced CD8:CD4 and CD8:FOXP3 T cell ratios. PitNET-derived chemokines facilitate macrophage, neutrophil and T cell recruitment into the tumours which can determine aggressive behaviour.

## Introduction

Pituitary neuroendocrine tumours (PitNETs) can be associated with significant morbidity due to mass effects on surrounding tissues and/or excessive or low hormone secretion [3, 46]. Twenty to 45% are invasive and may exhibit aggressive behaviour with cavernous sinus invasion and high Ki-67, and are often refractory to conventional treatment with poorer outcomes and higher recurrence rates [15, 18, 53, 82].

The tumour microenvironment (TME) consists of neoplastic, immune and stromal cells together with enzymes, growth factors and cytokines within the extracellular matrix [5–7, 11, 24]. Chemokines produced by the neoplastic cells contribute to trafficking and modulation of immune cells, angiogenesis and tumour invasion [5, 89]. Macrophages are a major component of the TME and are characterised by a spectrum of markers representing classically-activated macrophages (known as M1-like macrophages as one end of a spectrum) characterised by, among others, HLA-DR positivity [42, 44, 71], and alternatively-activated macrophages (representing another end, known as M2-like macrophages) characterised by, among others, CD163 positivity [27, 44, 81]. HLA-DR+ (M1-like) macrophages have an anti-tumoural effect [27, 60], whereas CD163+ (M2-like) macrophages are associated with tumour initiation, progression, invasion and angiogenesis [42, 44, 66, 73, 87]. Although the M1 and M2 macrophage nomenclature has been suggested to be replaced by more nuanced nomenclature [55], we use these forms when relating to previous literature [42, 44, 66, 73, 87]. Tumour-infiltrating CD8+ T lymphocytes are usually beneficial to the host as they initiate cytotoxic cascades against tumour cells; CD4+ T helper 1 cells are associated with good outcomes, whereas CD4+ T helper 2 and FOXP3+ T regulatory cells are immunosuppressive [6, 9]; B lymphocytes are often associated with a good prognosis, but an immunosuppressive B cell population has also been described [4, 49, 59, 69]. Neutrophils orchestrate responses against tumour cells, however, their role in tumour biology also includes detrimental effects [16, 61].

TME elements influence PitNET aggressiveness, rendering tumours larger [37, 91], more proliferative [8, 52] and invasive [38, 62, 88, 90]. Studying the TME may provide novel insights into PitNET biology and may lead to development of novel therapies, such as the successful ipilimumab and nivolumab combined treatment in a patient with an ACTH-secreting pituitary carcinoma [35]. Previous studies have assessed CXCL12 [8, 19, 33, 48, 58, 88, 91], IL-8 [23, 76, 84] and immune cells [6, 37, 38, 52, 85] in PitNETs. However, the crosstalk between the cytokine network and immune cells in determining the PitNET phenotype has not been previously comprehensively explored. In this study we aimed to characterise the cytokine network and the immune cell infiltrates within the TME of PitNETs.

## Materials and methods

### Human PitNET samples

Fresh human PitNET tissues from 24 patients (8 somatotropinomas and 16 clinically non-functioning PitNETs (NF-PitNETs), including 13 gonadotropinomas, 1 silent corticotropinoma and 2 null cell PitNETs) were obtained at the time of transsphenoidal surgery (Table 1), and a fragment was processed for primary culture. This study was approved by the local Ethics Committee (MREC No. 06/Q0104/133) and patients gave written informed consent. Normal pituitary (NP) autopsy samples collected 4–24 h post-mortem from subjects (age 18-81yr) with no known endocrine, immune or malignant disease (*n* = 5).

**Table 1:** Baseline clinicopathological features of the 24 patients with PitNETs

		Total of PitNETs (*n* = 24)
Gender *[n (%)]*		
Male		16 (66.7%)
Female		8 (33.3%)
Current age (yr) *[mean ± standard deviation (SD)]*		51.9 ± 15.1
Age at diagnosis (yr) *[mean ± SD]*		48.8 ± 15.5
Clinical diagnosis *[n (%)]*		
Acromegaly		8 (33.3%)
NF-PitNET		16 (66.7%)
Hyperprolactinaemia at diagnosis *[n (%)]*		8 (33.3%)
Headache *[n (%)]*		8 (33.3%)
Visual Impairment *[n (%)]*		13 (54.2%)
Hypopituitarism at diagnosis *[n (%)]*		11 (45.8%)
Macroadenoma *[n (%)]*		24 (100%)
Suprasellar extension *[n (%)]*		24 (100%)
Cavernous sinus invasion *[n (%)]*		10 (41.7%)
Ki-67 ≥ 3% *[n (%)]*		5 (20.8%)
Mean number of treatments *[mean ± SD]*		1.6 ± 0.9
Mean number of surgeries *[mean ± SD]*		1.2 ± 0.5
Reoperation *[n (%)]*		5 (20.8%)
Hypopituitarism at last follow-up *[n (%)]*		14 (58.3%)
Active disease at last follow-up *[n (%)]*		4 (16.7%)
Follow-up duration (yr) *[mean ± SD]*		2.5 ± 9.1
Pre-operative haematological parameters *[mean ± SD]*		
Red cell count (10^12^/L)	*[Normal range (NR): M 4.4–5.8 / F 3.95–5.15]*	4.58 ± 0.46
Haemoglobin (g/L)	*[NR: M 130–170 / F 115–155]*	132.46 ± 12.47
Haematocrit (%)	*[NR: M 37–50 / F 33–45]*	40.24 ± 3.79
White cell count (10^9^/L)	*[NR: 3.0–10.0]*	7.08 ± 3.39
Neutrophil count (10^9^/L)	*[NR: 2.0–7.5]*	3.73 ± 1.51
Lymphocyte count (10^9^/L)	*[NR: 1.2–3.65]*	2.71 ± 2.25
Monocyte count (10^9^/L)	*[NR: 0.2–1.0]*	0.44 ± 0.13
Eosinophil count (10^9^/L)	*[NR: 0.0–0.4]*	0.17 ± 0.11
Basophil count (10^9^/L)	*[NR: 0.0–0.1]*	0.03 ± 0.02
Platelet count (10^9^/L)	*[NR: 150–400]*	234.96 ± 62.18

### Immunohistochemical analysis and evaluation

Immunostains were performed on 4 μm paraffin-embedded tissue sections using Ventana Discovery DAB Map System (Ventana, Illkirch, France). Slides were deparaffinised and processed for antigen retrieval for 30 min with cell conditioning solution CC1 (Ventana), which is a Tris base buffer (pH~ 9). After blocking with Blocker D solution (Ventana), slides were incubated with primary antibody for 60 min (Additional file 4: Table S1) and then with the universal secondary antibody (Ventana) for 20 min. Slides were counterstained with haematoxylin. A negative control, where primary antibody was omitted, was included per experiment. Immunohistochemical studies assessed macrophages using CD68, CD163 and HLA-DR [42, 44, 71, 81], lymphocytes using CD8 for cytotoxic T cells, CD4 for T helper cells, FOXP3 for T regulatory cells and CD20 for B cells. Neutrophils were assessed with neutrophil elastase and endothelial cells with CD31 as well as by their location and morphology. Slides were scanned and analysed with Pannoramic Scanner and Viewer Software (3DHISTECH, Budapest, Hungary). Full stained slides were inspected at low magnification (5x and 10x magnification) in order to identify “hot spots” areas, i.e. areas with high abundance of immunopositive cells within the tumoural tissue in PitNET slides and within the normal pituitary tissue in NP slides. All selected areas were first verified on the respective haematoxylin and eosin slides, to ensure that cell counting was correctly performed in tumoural or normal pituitary tissue respectively. We excluded intra-vascular and non-adenomatous immunopositive cells. Immunopositive cells were counted in 5 different “hot spots” high power field (HPF) using the ImageJ software (National Institutes of Health, USA). Counterstained nuclei identifying tumour cells were also counted, and the data were expressed as percentage of immunopositive immune cells relatively to the total number of tumour cells per HPF. Vessels (stained for CD31) were manually counted in 3–5 different “hot spot” fields (20x magnification) allowing the estimation of microvessel density (number of vessels per HPF), and the vessels contour was manually traced using the ImageJ software to obtain an estimation of the total microvessel area (μm^2^), as described previously [78].

### Pre-operative haematological data collection

Blood samples from each patient were routinely taken within 7–10 days before the pituitary surgery as part of the pre-operative work-up protocol. All the serum full blood counts were performed in a certified National Health Service laboratory in a standardised manner on a Sysmax automated counter.

### RNA in situ hybridisation with RNAscope

IL-8 and CCL2 and chemokine receptors CXCR2 and CCR5 were detected using the RNAscope 2.5 HD Duplex Chromogenic Assay (Advanced Cell Diagnostics, ACD, USA), according to the manufacturer’s protocols. Briefly, 4 μm paraffin-embedded PitNET tissue sections were baked at 60 °C for 90 min, deparaffinised, and then boiled with pre-treatment retrieval reagent for 15 min. Protease digestion was performed at 40 °C for 30 min, followed by hybridisation for 2 h at 40 °C with Probe mix according to 1:50 ratio of C2 to C1 probes: mix IL-8 (ACD, cat. no. 310381-C2) and CXCR2 (ACD, cat. no. 468411), and mix CCR5 (ACD, cat. no. 601501-C2) and CCL2 (ACD, cat. no. 423811). Hybridisation signals were amplified and visualised with RNAscope 2.5 HD Duplex Chromogenic Assay reagents. Cell nuclei were counterstained with haematoxylin, and slides were mounted with VectaMount mounting medium (Vector Laboratories, cat. no. H-5000). Probe-DapB (ACD) was used as negative control. Slides were scanned and analysed with Pannoramic Scanner and Viewer Software (3DHISTECH, Budapest, Hungary).

### Primary culture

Fresh PitNET tissue was collected in high glucose Dulbecco’s Modified Eagle’s Medium (DMEM, Sigma, Gillingham, UK, cat. no. D6429) supplemented with 10% heat-inactivated foetal bovine serum (FBS, Gibco, Loughborough, UK, cat. no. 16000044) and 0.5% gentamicin (Sigma, cat. no. G1397). Tissue was washed with magnesium and calcium-free Phosphate Buffered Saline (PBS) (Sigma, cat. no. D8537), cut into small pieces and incubated for 45 min at 37 °C in 10 times diluted Trypsin-EDTA 0.05% (1X) Phenol Red (Gibco, cat. no. 25300054) with frequent pipetting allowing effective cell dispersion. Trypsin digestion was stopped by adding complete medium, cells were transferred to a tube and allowed to stand for 10 min for sedimentation of undigested debris. Supernatants containing tumour cells were transferred to a separate tube, centrifuged at 800 g for 5 min, and gently re-suspended in 1 mL complete medium. Viable cells were assessed with Tryptan Blue Solution (Sigma, cat. no. T8154) and 2 × 10^6^ cells were seeded in complete medium in a well coated with Poly-L-lysine (Sigma, cat. no. P4707) from a 6-well plate when viability was > 90%. Cells were incubated overnight at 5% CO_2_ at 37 °C. The next day plates were examined under the microscope, complete medium was aspirated, cells were carefully washed 3 times with PBS, and then 1 mL serum-free medium was added. After incubation for 24 h, supernatants were collected to clean tubes and placed immediately on ice to avoid cytokine degradation. Tubes were centrifuged at 10,000 rpm for 10 min at 4 °C to remove debris, and supernatants were collected and stored in − 80 °C for 3–6 months until being assayed in duplicate with the human Millipore MILLIPLEX cytokine 42-plex array.

### Cell culture

Rat pituitary somatomammotroph GH3 cell line, obtained from European Collection of Authenticated Cell Cultures, and the murine RAW 264.7 macrophages (kind gift from Dr. Giulia Marelli, Barts Cancer Institute) were incubated at 5% CO_2_ at 37 °C and cultured in high glucose DMEM supplemented with 10% FBS and 0.5% gentamycin. Once cells were 70–90% confluent, they were passaged after washes with magnesium- and calcium-free PBS, and mobilisation with Trypsin-EDTA 0.05% (1X) Phenol-Red for GH3 cells or with Accutase® solution (Sigma, cat. no. A6964) for RAW 264.7 macrophages. Once cells were detached (confirmed by light microscopy), cell suspension was put into new flasks or spun (3 min, 1200 g) and re-suspended in medium to be further used in in vitro experiments.

GH3 cell-conditioned medium (CM) was generated by seeding 5 × 10^6^ GH3 cells in T75 culture flasks for 72 h in 10 mL complete medium. Macrophage-CM was generated from 5 × 10^6^ RAW 264.7 macrophages in T75 culture flasks for 24 h in 10 mL complete medium (−PMA_Raw.CM) or stimulated with 5 nM of Phorbol 12-Myristate 13-Acetate (PMA) (Sigma, cat. no. P8139) in 10 mL complete medium (+PMA_Raw.CM).

Cell culture supernatants for cytokine array were generated by seeding 5 × 10^5^ GH3 cells in 12-well culture plates for 24 h in serum-free medium conditions at baseline and after treatment with RAW 264.7 macrophage-CM. Supernatants were carefully transferred to clean tubes, centrifuged at 10,000 rpm for 10 min in 4 °C to remove debris, and then supernatants were collected and stored in − 80 °C for 3–6 months until being assayed.

### Human and rat Millipore MILLIPLEX cytokine arrays

Cytokine arrays on human PitNET-derived supernatants were performed by Eve Technologies (Calgary, Alberta, Canada), according to their protocol by using Bio-Plex™ 200 system (Bio-Rad Laboratories, Inc., Hercules, CA, USA), and human cytokine/chemokine array with IL-18 (HD42) kit (Millipore, St. Charles, USA). This array measured 42 different cytokines, chemokines and growth factors in the same sample: G-CSF, GM-CSF, IFNα2, IFNγ, IL-1α, IL-1β, IL-1ra, IL-2, IL-3, IL-4, IL-5, IL-6, IL-7, IL-8, IL-9, IL-10, IL-12(p40), IL-12(p70), IL-13, IL-15, IL-17A, IL-18, CXCL1, CXCL10, CCL2, CCL3, CCL4, CCL5, CCL7, CCL11, CCL22, CX3CL1, sCD40L, Flt-3 L, PDGF-AA, PDGF-BB, TGF-α, TNF-α, TNF-β, VEGF-A, EGF and FGF-2.

Millipore MILLIPLEX arrays on supernatants from rat GH3 cells were also performed by Eve Technologies, using a different species-specific kit array: rat cytokine/chemokine array 27-plex (RD27) (Millipore) able to measure 27 different cytokines, chemokines and growth factors in the same sample: G-CSF, GM-CSF, IFNγ, IL-1α, IL-1β, IL-2, IL-4, IL-5, IL-6, IL-10, IL-12(p70), IL-13, IL-17A, IL-18, CXCL1, CXCL2, CXCL10, CCL2, CCL3, CCL5, CCL11, CX3CL1, TNF-α, VEGF, EGF, Leptin and LIX.

### Invasion, migration and morphology studies

Invasion assays were carried out using the BioCoat Matrigel Invasion Chambers with 8 μm pores (24-well insert; BD Biosciences, CA, USA, cat. no. 354480). Invasion chambers were hydrated for 2 h with 500 μl of serum-free medium at 37 °C. After Matrigel rehydration, 750 μl of macrophage-CM or complete medium was added to the lower chamber as chemoattractant and 2.5 × 10^4^ GH3 cells in 500 μl serum-free medium were added to the upper chamber and incubated at 37 °C. After 72 h, invading cells through Matrigel were fixed in 100% methanol and stained with 2% Giemsa blue (Sigma-Aldrich, MO, USA, cat. no. G5637-5G). Total number of invading cells per chamber were counted, and normalised to invading cells towards complete medium. Macrophage chemotaxis were evaluated by Transwell BioCoat Migration insert plates with 8 μm pores (24-well insert; Corning Fisher Scientific, USA, cat. no. 354578) following a similar protocol as described for invasion assay. Migration and invasion studies were run in duplicates and were repeated at least 3 times. Morphological changes were assessed, as previously described [10], by measuring 6 different shape parameters using ImageJ: area (area of selection in calibrated square units, μm^2^); perimeter (μm); Feret’s diameter (longest distance between any 2 points along selection boundary); roundness (representing shape, 4 × [Area] / π × [Major axis]^2^, with value of 1 for a circle and 0 for very elongated shapes); circularity (representing perimeter smoothness, 4π × [Area][Perimeter]^2^, with value of 1 indicating a perfect circle and value close to 0 indicating elongated shape) and solidity (value of 1 indicating more stiffness and less deformable cells). Per treatment condition, 5 images at 40x were taken and 15 cells were measured per image, thus 75 cells were analysed per experiment (minimum of 3 experiments were done).

### Immunocytochemistry

GH3 cells (5 × 10^4^) were plated on 15 mm coverslips placed in 12-well plates. After overnight attachment, GH3 cells were treated with -PMA_Raw.CM, +PMA_Raw.CM or complete medium for 24 h. Cells were fixed in 4% paraformaldehyde for 15 min at room temperature, following washes with PBS cells were permeabilised with 0.1% Triton X-100 in PBS for 5 min at 4 °C. Cells were blocked in 1% bovine serum albumin for 30 min at room temperature, and then incubated with primary antibodies (listed in the Additional file 4: Table S1) followed by a 30 min incubation with secondary conjugated antibodies (Alexa Fluor 568-conjugated goat anti-mouse IgG, Alexa Fluor 488-conjugated donkey anti-mouse IgG and Alexa Fluor 488-conjugated donkey anti-rabbit IgG; 1:1000; Molecular Probes, Invitrogen). Coverslips with stained cells were mounted with Fluoroshield with DAPI mounting medium (Sigma, cat. no. F6057). Stained slides were visualised on confocal microscope LSM 880 Zeiss and images taken at 63x magnification. E-cadherin and ZEB1 fluorescent intensities were quantified using the software Carl Zeiss Zen Blue Edition v2.3.

### Real-time quantitative polymerase chain reaction (RT-qPCR)

RNA from GH3 cells and RAW 264.7 macrophages was extracted using Qiagen’s RNeasy micro kit (cat. no. 74004) according to manufacturer’s protocol. RNA samples were assessed by NanoDrop ND-1000 spectrophotometer (NanoDrop Technologies, Rockland, DE, USA). Complementary DNA was synthesised from 1 μg of RNA using High-Capacity cDNA Reverse Transcription Kit (Thermofisher Scientific, cat. no. 4374966) following the manufacturer’s protocol. RT-qPCR reactions were prepared using Brilliant III Ultra-Fast SYBR Green QPCR Master Mix (Agilent Technologies, Palo Alto, CA, USA, cat. no. 600882) and run with Thermal Cycler with MxPro software (Agilent) using a 2-step programme: pre-incubation 3 min at 95 °C, then 40 cycles of 20s at 95 °C and 20s at 60 °C. Cycle threshold (CT) values were analysed with ∆∆CT quantification method. Target gene expression was normalised to GAPDH expression used as internal control. Primers sequence (Sigma-Aldrich) were as follows: CCR5 forward 5′-GTATGTCAGCACCCTGCCAA-3′, reverse 5′-GAGCAGGAAGAGCAGGTCAG-3′; CX3CR1 forward 5′-CCATCTGCTCAGGACCTCAC-3′, reverse 5′-CACCAGACCGAACGTGAAGA-3′; MMP9 forward 5′-CTTGAAGTCTCAGAAGGTGGATC-3′, reverse 5′-CGCCAGAAGTATTTGTCATGG-3′.

### Affymetrix microarray analysis and xCell deconvolution

Total RNA from a different set of 7 human sporadic PitNET samples (3 somatotropinomas and 4 NF-PitNETs) and 5 NPs was isolated using the Qiagen’s RNeasy micro kit and target labelling and hybridisation were performed using Affymetrix GeneChip 3′ IVT Express Kit (Affymetrix, Santa Clara, CA, USA) according to manufacturer’s instructions (samples described in detail in [10]). Microarray data have been deposited with the National Center for Biotechnology Information Gene Expression Omnibus (www.ncbi.nlm.nih.gov/geo, accession number GSE63357). For deconvolution, we used the gene-signature webtool method xCell [2, 56], which inferred different immune and stromal cells from microarray expression data giving a score per cell type. This tool uses the M1 and M2 macrophage nomenclature.

### Statistical analysis

Statistical analyses were carried out using the SPSS statistical software v20 (IBM, USA) and GraphPad v6 (Prism, USA). Mann Whitney U test, one-way or two-way ANOVA tests with post-hoc comparison tests were applied as appropriate. Correlations between continuous variables were determined by Pearson correlation coefficient *r. p* values < 0.05 were considered significant.

## Results

### Pituitary tumour cells release chemokines, with NF-PitNETs secreting higher amounts than somatotropinomas

**Fig. 1:**
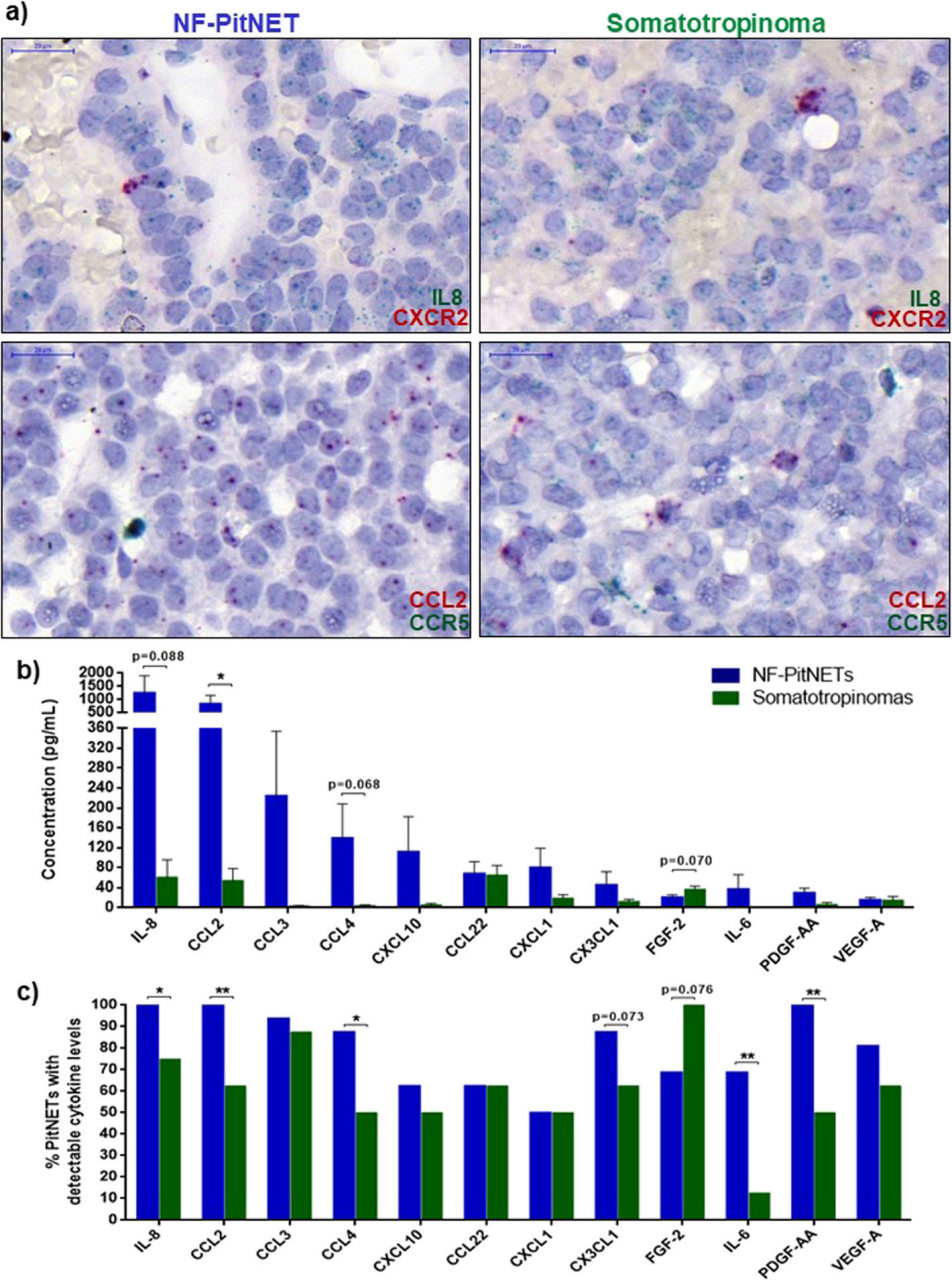
**a** RNAscope staining of IL8 (green)-CXCR2 (red) and CCL2 (red)-CCR5 (green) mRNA in a NF-PitNET and a somatotropinoma. CCL2 and IL-8 are mainly expressed in pituitary tumour cells, while the chemokine receptors are strongly expressed in scattered perivascular cells (morphologically distinct from tumour cells), likely corresponding to immune cells. Scale bar 20 μm. **b** Cytokine secretome from NF-PitNETs (*n* = 16) and somatotropinomas (*n* = 8). Data are shown for the top 12 secreted proteins as mean ± standard error of the mean. *, <0.05 (Mann Whitney U test). **c** Percentage of NF-PitNETs (*n* = 16) and somatotropinomas (*n* = 8) with detectable cytokine levels, i.e. concentration above the lowest standard curve and serum-free medium quantification. Data are shown as percentage and for the top 12 secreted proteins as identified by the Millipore MILLIPLEX assay in primary culture supernatants. *, < 0.05, **, < 0.01 (Chi-squared test)

In order to identify the most relevant cytokines derived from human PitNETs, we established primary cultures from 24 PitNETs. All tumours were larger than 1 cm in diameter, 10 had cavernous sinus invasion and 5 had Ki-67 ≥ 3% (Table 1). We assessed 42 different cytokines in fresh tumour culture supernatants (Additional file 5: Table S2). The cytokine array identified IL-8, CCL2, CCL3, CCL4, CXCL10, CCL22, CXCL1 and CX3CL1 as the main PitNET-derived cytokines (Table 2), all chemokines specialised in immune cell recruitment [5]. Ninety percent of PitNETs secreted IL-8, CCL2 and CCL3, while CXCL1 was secreted by 50% of the tumours (Table 2). RNAscope data showed that CCL2 and IL-8 are mainly synthesised by pituitary tumour cells, while these have low expression of chemokine receptors; chemokine receptors were, in turn, strongly expressed in scattered perivascular cells, morphologically distinct from tumour cells, likely corresponding to immune cells (Fig. 1a).

**Table 2:** Top 12 highly secreted cytokines/chemokines/growth factors in the human PitNETs-derived cell culture supernatants (*n* = 24)

Cytokine/ Chemokine/ Growth factor	Mean concentration (pg/mL) ± SEM	Serum-free medium (pg/mL)	n (%) of PitNETs with detectable cytokine
IL-8	854.18 ± 445.79	7.06	22 (91.7%)
CCL2	578.03 ± 222.66	4.00	21 (87.5%)
CCL3	150.55 ± 88.22	0	22 (91.7%)
CCL4	94.25 ± 47.20	3.09	18 (75.0%)
CXCL10	76.67 ± 47.58	0	14 (58.3%)
CCL22	67.25 ± 16.74	20.78	15 (62.5%)
CXCL1	60.30 ± 26.14	20.78	12 (50.0%)
CX3CL1	35.14 ± 17.12	6.73	19 (79.2%)
FGF-2	26.65 ± 4.11	0	19 (79.2%)
IL-6	24.90 ± 19.27	0	12 (50.0%)
PDGF-AA	22.36 ± 6.78	0.12	20 (83.3%)
VEGF-A	15.85 ± 4.06	0	18 (75.0%)

PitNETs-derived supernatants collected at 24 h on serum-free medium conditions and cytokine secretome determined with the human Millipore MILLIPLEX cytokine 42-plex array. Data are shown as mean concentration (pg/mL) ± standard error of the mean (SEM). Right column shows the number and percentage of PitNETs with detectable cytokine levels, i.e. cytokine concentration above the lowest standard curve point and serum-free medium quantification

NF-PitNETs secreted higher amounts of cytokines/chemokines than somatotropinomas, especially CCL2 (16x more), IL-8 (25x more) and CCL4 (27x more), except for FGF-2 which was found in higher concentrations in somatotropinoma supernatants (Fig. 1b). NF-PitNETs were more often secreting IL-8, CCL2, CCL4, IL-6 and PDGF-AA than somatotropinomas (Fig. 1c). Secretome differences between NF-PitNETs and somatotropinomas were not explained by pre-operative somatostatin analogue treatment, as there were no secretome differences between pre-treated and non-pre-treated somatotropinomas (Additional file 1: Figure S1a). There were also no secretome differences between sparsely and densely granulated somatotropinomas (Additional file 1: Figure S1b). The PitNET-derived cytokine secretome was not associated per se with cavernous sinus invasion, elevated Ki-67 or presence of hypopituitarism at diagnosis (data not shown).

### Immune infiltrates differ between PitNETs and NPs

**Fig. 2:**
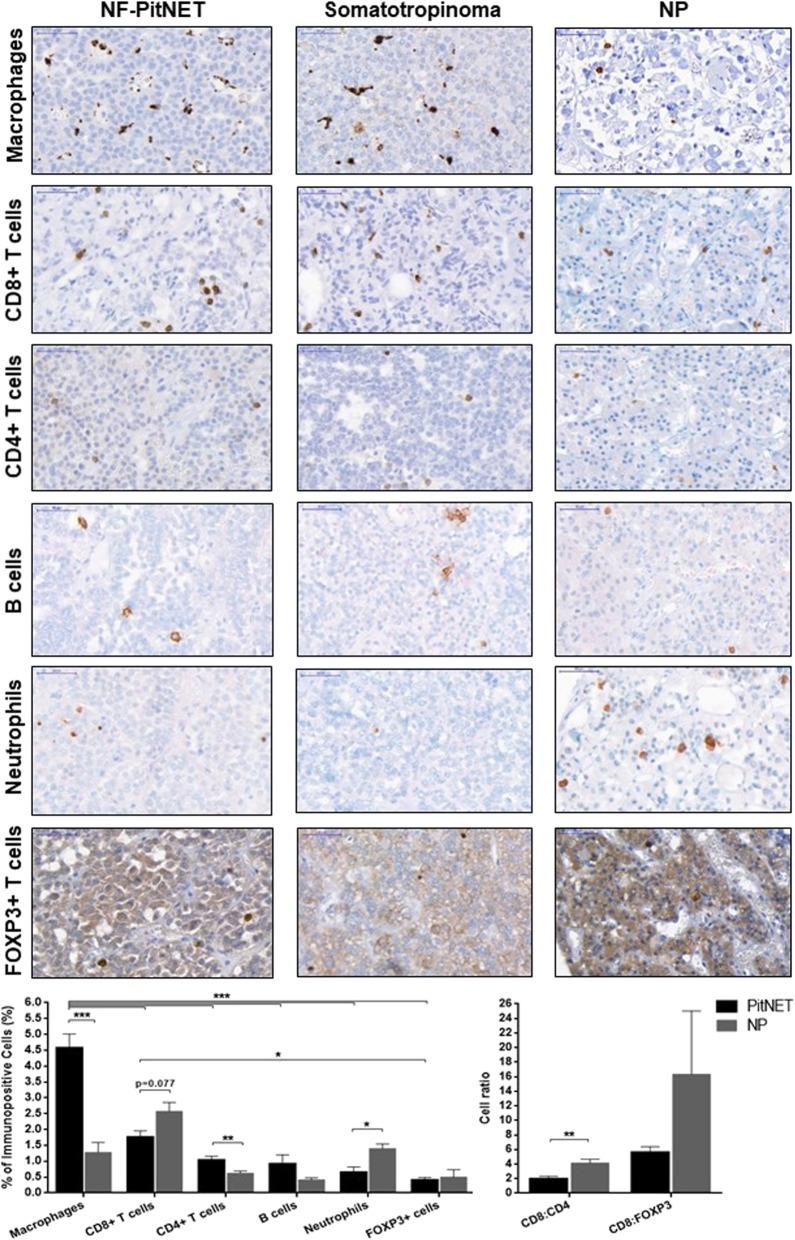
Immunohistochemical analysis of immune cells in PitNETs and normal pituitary (NP). Immune cells analysed: macrophages (CD68+), cytotoxic T lymphocytes (CD8+), T helper lymphocytes (CD4+), T regulatory cells (FOXP3+), B cells (CD20+) and neutrophils (neutrophil elastase+). Data are shown as mean ± standard error of the mean for percentage of immune cells compared to the total number of tumour cells, and for CD8:CD4 or CD8:FOXP3 cell ratios. Representative images are shown for NF-PitNET, somatotropinoma and NP. Scale bar 50 μm. PitNETs, *n* = 24; NPs, *n* = 5. *,< 0.05, **,< 0.01, ***,< 0.001 (two-way ANOVA with Bonferroni multiple comparison test for immunopositive cell comparative analysis; Mann Whitney U test for cell ratio comparative analysis)

PitNETs contained more CD68+ macrophages (4.6 ± 0.4 vs 1.2 ± 0.2%, *p* < 0.001) and CD4+ T cells (1.0 ± 0.1 vs 0.6 ± 0.1%, *p* = 0.005), but fewer neutrophils (0.7 ± 0.2 vs 1.4 ± 0.1%, *p* = 0.047) and a trend for fewer CD8+ T cells (1.8 ± 0.2 vs 2.6 ± 0.3%, *p* = 0.077), with a significant 2-fold decrease in the CD8:CD4 cell ratio compared to NPs (Fig. 2). There were no significant differences in B and FOXP3+ T cells content (Fig. 2).

**Fig. 3:**
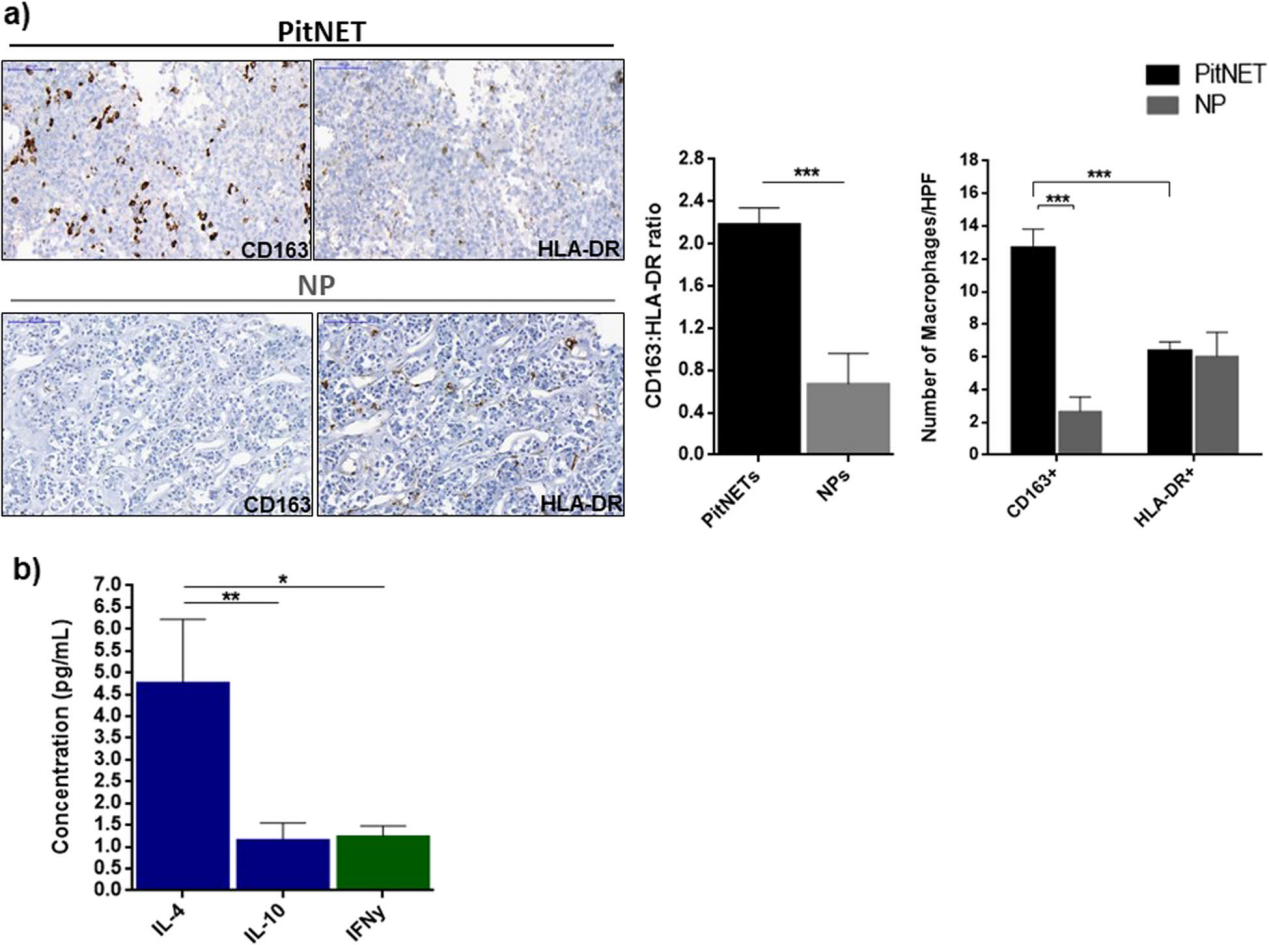
**a** Immunohistochemical analysis of CD163+ and HLA-DR+ macrophages in PitNETs and normal pituitary (NP). Data are shown as mean ± standard error of the mean (SEM) for CD163:HLA-DR ratio and for the number of CD163-positive and HLA-DR-positive cells per high power field (HPF). Representative images are shown for a PitNET and NP. Scale bar 50 μm. PitNETs, *n* = 24; NP, *n* = 5. ***, < 0.001 (Mann Whitney U test). **b** Macrophage-polarising cytokines in PitNET culture supernatants. Supernatants were collected at 24 h in serum-free medium conditions and cytokine secretome determined with the human Millipore MILLIPLEX cytokine 42-plex array. Results are shown as mean ± SEM for IL-4 and IL-10 (M2-polarising cytokines, blue bars) and IFNγ (M1-polarising cytokine, green bar). *,< 0.05, **,< 0.01 (one-way ANOVA with Bonferroni multiple comparison test)

Macrophages are the most abundant immune cell type in PitNETs, while other immune cells are present in lower amounts (Fig. 2). Macrophages in PitNETs are predominantly CD163+, while HLA-DR+ macrophages predominate in NPs, resulting in a 3-fold increase in the CD163:HLA-DR macrophage ratio in PitNETs (Fig. 3a). These immunohistochemical findings were confirmed on a different set of samples [10] using gene expression analysis with xCell, with PitNETs having a higher score than NPs for macrophages (0.090 ± 0.016 vs 0.031 ± 0.013; *p* = 0.025) and CD163+ macrophages (0.042 ± 0.007 vs 0; *p* = 0.001) (Additional file 2: Figure S2). The CD163+ macrophage phenotype in PitNETs may be due, at least in part, to higher concentrations of PitNET-derived M2-polarising cytokines, as IL-4 levels were almost 5x higher than interferon-γ (M1-polarising cytokine) (Fig. 3b).

We observed significant correlations between infiltrating immune cell populations in PitNETs, namely between CD8+ and CD4+ T cells (*r* = 0.534; *p* = 0.007), CD8+ and FOXP3+ T cells (*r* = 0.504; *p* = 0.012), CD4+ T cells and neutrophils (*r* = 0.490; *p* = 0.05).

NF-PitNETs had more neutrophils than somatotropinomas (0.9 ± 0.1 vs 0.1 ± 0.1%, *p* = 0.002), but there were no differences regarding other immune cells, neither CD163:HLA-DR, CD8:CD4 or CD8:FOXP3 cell ratios (Additional file 6: Table S3). There were no significant differences in infiltrating immune cells among the different NF-PitNET types (Additional file 6: Table S3) or between sparsely and densely granulated somatotropinomas (data not shown).

### Tumour cell-derived cytokines attract immune cells into the TME

**Fig. 4:**
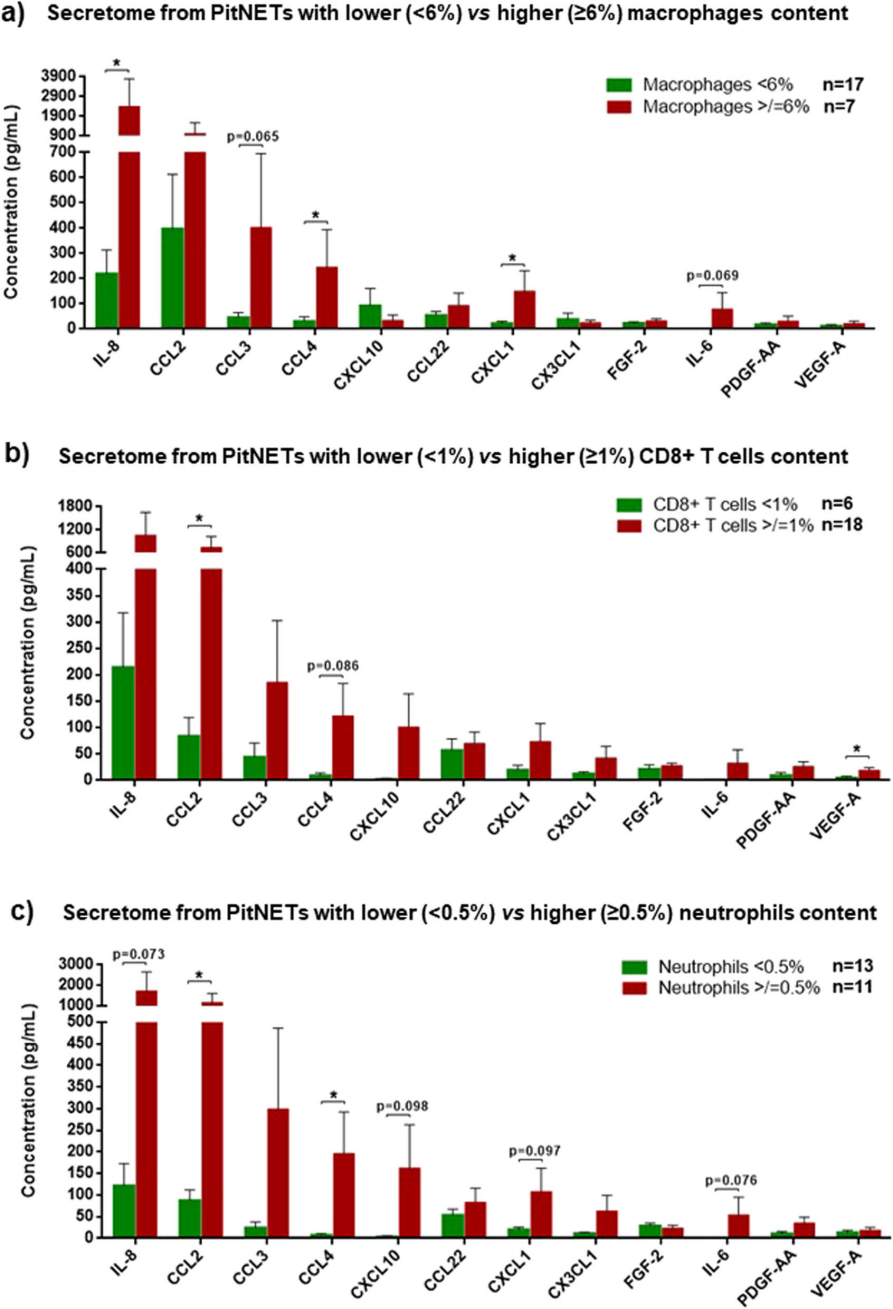
Cytokine secretome of PitNETs cell culture supernatants with lower vs higher content of macrophages (**a**), CD8+ T cells (**b**) and neutrophils (**c**). The cutoffs used to define low and high immune cell content were: 6% for macrophages, 1% for CD8+ T cells and 0.5% for neutrophils. Data are shown for the top 12 secreted proteins as mean ± standard error of the mean. *, < 0.05 (Mann Whitney U test)

PitNETs with higher macrophage content were associated to higher levels of IL-8 (*p* = 0.023), CCL2 (*p* = 0.216), CCL3 (*p* = 0.065), CCL4 (*p* = 0.036) and CXCL1 (*p* = 0.024) (Fig. 4a), chemokines known to promote macrophage chemotaxis [1, 5, 41, 43]. We observed higher CCL2 (p = 0.036), CCL4 (*p* = 0.086), CXCL10 (*p* = 0.134) and VEGF-A (*p* = 0.025) levels in the supernatants from PitNETs with higher CD8+ T cell contents (Fig. 4b). PitNETs with more neutrophils released higher levels of CCL2 (*p* = 0.033) and CCL4 (*p* = 0.044), cytokines known to attract neutrophils [16, 63, 92], and there was a noteworthy trend to release higher levels of chemokines involved in neutrophil chemotaxis (Fig. 4c), namely IL-8 (*p* = 0.073), CXCL1 (*p* = 0.097) and CXCL10 (*p* = 0.098). There were no significant associations between PitNET-derived cytokine secretome and infiltrating CD4+, FOXP3+ and B cells (data not shown).

PitNET-infiltrating immune cells did not correlate with circulating immune cell types, suggesting that immune infiltrates are subject to differential recruitment into the PitNET rather than altered bone marrow production (Additional file 3: Figure S3).

### Immune cells in the TME of PitNETs may determine tumour proliferation and angiogenesis

**Fig. 5:**
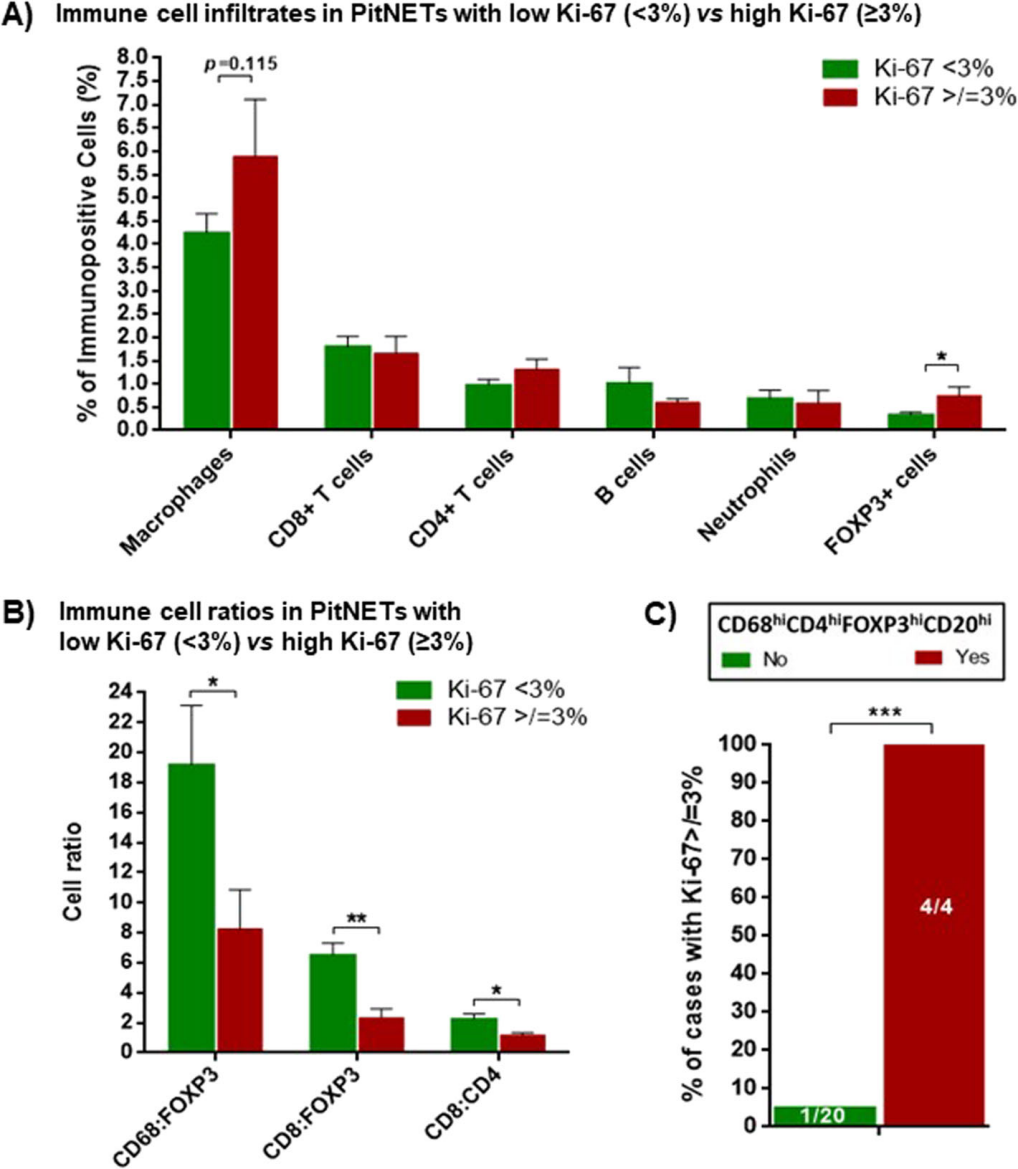
Immune cell infiltrates (**a**) and immune cell ratios (**b**) in PitNETs with lower (< 3%) vs higher (≥3%) Ki-67. PitNETs with lower Ki-67, *n* = 19; PitNETs with higher Ki-67, n = 5. *, < 0.05, **, <0.01 (Mann Whitney U test). **c**) Percentage of PitNETs with Ki-67 ≥ 3% according to the presence of a “deleterious immune infiltrate phenotype”, i.e. higher content of macrophages, CD4+ T helper lymphocytes, FOXP3+ T regulatory cells and B cells (CD68^hi^CD4^hi^FOXP3^hi^CD20^hi^). PitNETs with “deleterious immune infiltrate phenotype”, *n* = 4; PitNETs without “deleterious immune infiltrate phenotype”, *n* = 20. ***, <0.001 (Exact Fisher’s test)

**Fig. 6:**
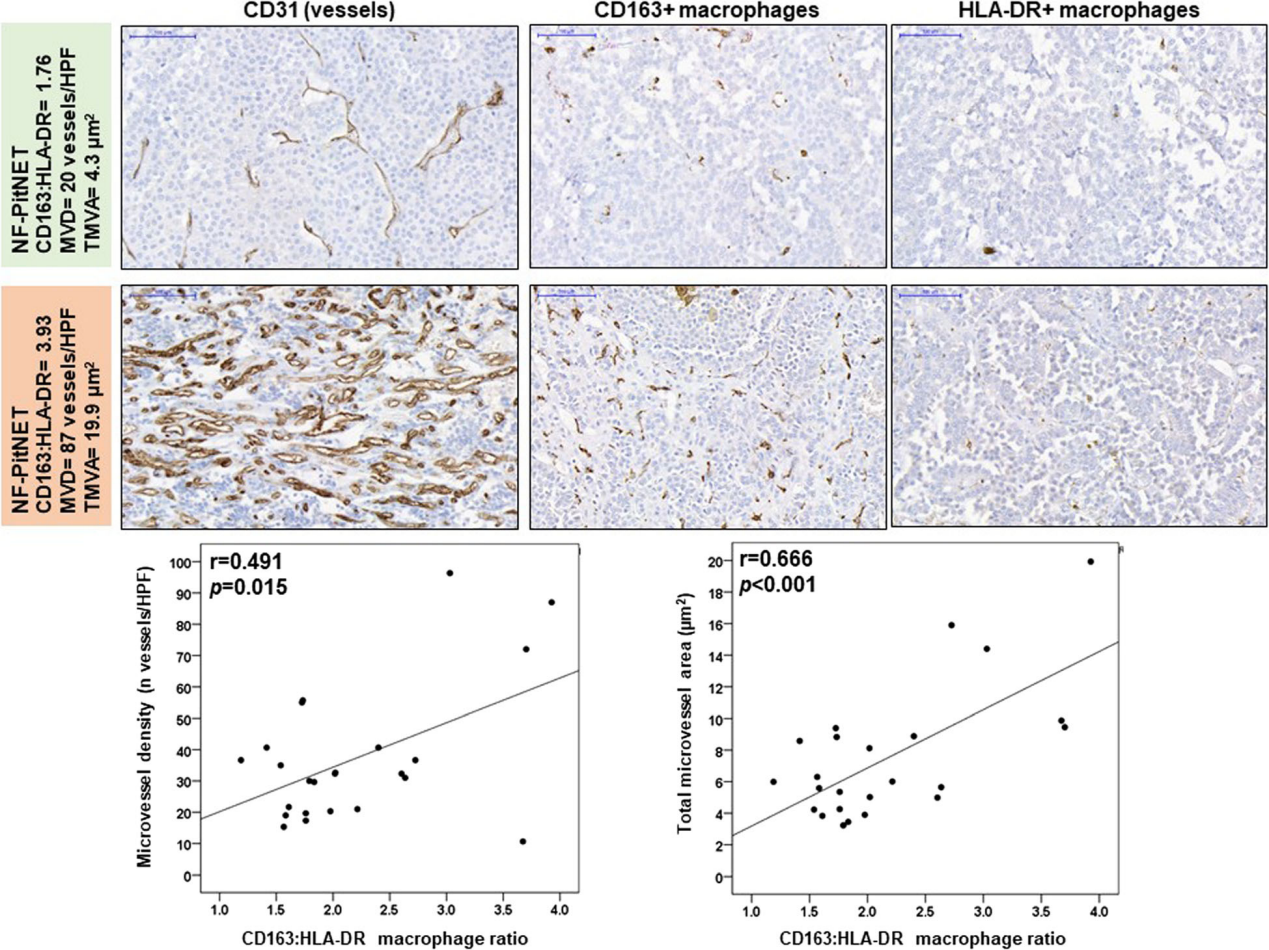
Microvessel density (MVD) or area (TMVA) correlation with CD163:HLA-DR macrophage ratio**.** Representative images are shown from samples with low and high CD163:HLA-DR ratio. Scale bar 100 μm, *n* = 24. *P* values were determined by the Pearson correlation

PitNETs with higher proliferative index (Ki-67 ≥ 3%) had lower CD8:CD4 ratio, as well as lower CD8:FOXP3 and CD68:FOXP3 ratios as a result of increased infiltration of FOXP3+ T cells (0.7 ± 0.2 vs 0.3 ± 0.6%; *p* = 0.013) (Fig. 5a-b). All PitNETs with “deleterious immune infiltrate phenotype”, i.e. higher content of macrophages, T helper lymphocytes, FOXP3+ T regulatory cells and B cells (CD68^hi^CD4^hi^FOXP3^hi^CD20^hi^) had a Ki-67 ≥ 3% (Fig. 5c). There were no differences between PitNETs with or without cavernous sinus invasion regarding immune cell content or cell ratios (data not shown). The CD163:HLA-DR macrophage ratio was positively correlated with microvessel density (*p* = 0.015) and area (*p* < 0.001) (Fig. 6).

### GH3 cell-derived factors increase macrophage chemotaxis and alter their morphology

**Fig. 7:**
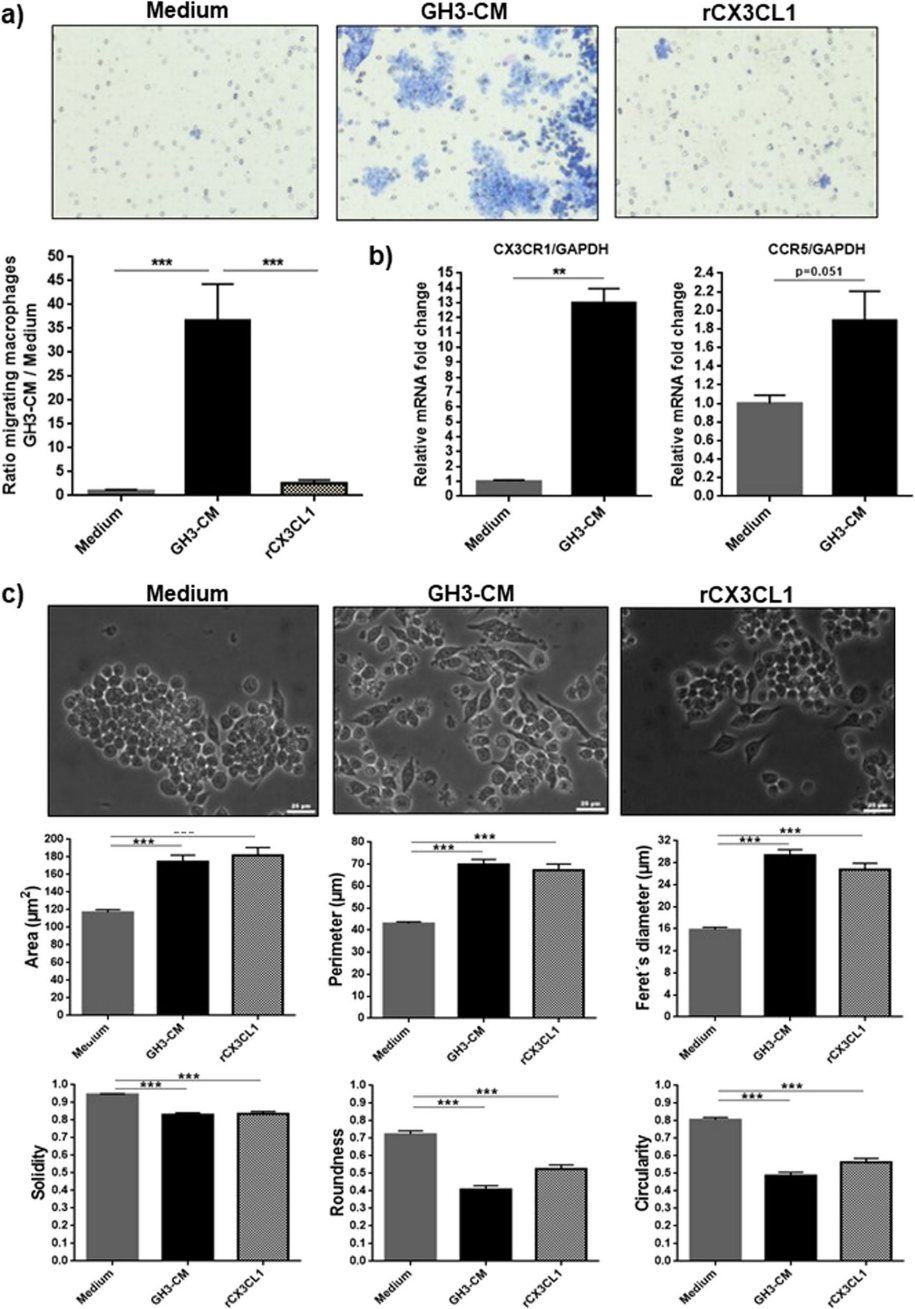
**a** Transwell chemotaxis assay performed on RAW 264.7 macrophages towards complete medium, GH3-CM and recombinant CX3CL1 (rCX3CL1) at concentration 100 ng/mL for 72 h. Data are shown as mean ± standard error of the mean (SEM) for the ratio of migrated RAW 264.7 macrophages towards GH3-CM or rCX3CL1 in relation to migrated macrophages in complete medium. *n* = 6. ***, <0.001 (one way-ANOVA with Bonferroni multiple comparison test). **b** CX3CR1 and CCR5 expression in RAW 264.7 macrophages determined by RT-qPCR after treatment with GH3-CM for 24 h vs complete medium. Data are shown as mean ± SEM for CX3CR1 or CCR5 relative fold change expression to GAPDH, determined by the ∆∆CT method. *n* = 3. **, <0.01 (Mann Whitney U test). c Morphological evaluation of RAW 264.7 macrophages after treatment for 72 h with complete medium (*n* = 3), GH3-CM and rCX3CL1 at concentration of 100 ng/mL. Data are shown as mean ± SEM for the 6 morphological parameters evaluated by Image J: cell area (μm^2^), Feret’s diameter (μm), solidity (0–1), perimeter (μm), roundness (0–1) and circularity (0–1). Per experiment 75 cells were analysed, with a minimum of 3 experiments per treatment condition. Scale bar 25 μm. ***, <0.001 (one-way ANOVA with Bonferroni multiple comparison test)

To study the interactions between pituitary tumour cells (GH3 mammosomatotroph tumour cell line) and macrophages (RAW 264.7 macrophage cell line), we established an in vitro model using CM from each of the cell line as a chemoattractant agent for the other. To investigate the role of GH3 cell-derived factors in macrophage chemotaxis, we performed a transwell migration assay, observing a remarkable 36-fold increase in macrophage migration towards GH3-CM in comparison to complete medium or recombinant CX3CL1 (Fig. 7a). CX3CL1 was used as positive control, as this was the chemokine with the highest concentration in GH3 supernatants (Additional file 7: Table S4), and has a recognised chemoattractant effect on RAW 264.7 macrophages [22]. Immune cell chemotaxis depends not only on tissue chemokine gradient, but also on chemokine receptor expression in trafficking cells [5]. GH3-CM increased more than 12x the expression of CX3CR1 (receptor with specific affinity for CX3CL1 and highly expressed in RAW 264.7 macrophages [22]), as well as the expression of CCR5 (*p* = 0.051) (Fig. 7b). Thus, the GH3-CM macrophage chemoattractant effect can be explained, at least in part, by upregulation of chemokine receptor expression in RAW 264.7 macrophages. After GH3-CM treatment, macrophages showed morphological changes typical of activated macrophages: increase in cell area, perimeter, Feret’s diameter and spindle-shaped morphology [42, 44] and decrease in solidity, roundness and circularity (Fig. 7c), representing cells with enhanced migration phenotype.

### Macrophage-derived factors affect the behaviour and invasiveness of GH3 cells

**Fig. 8:**
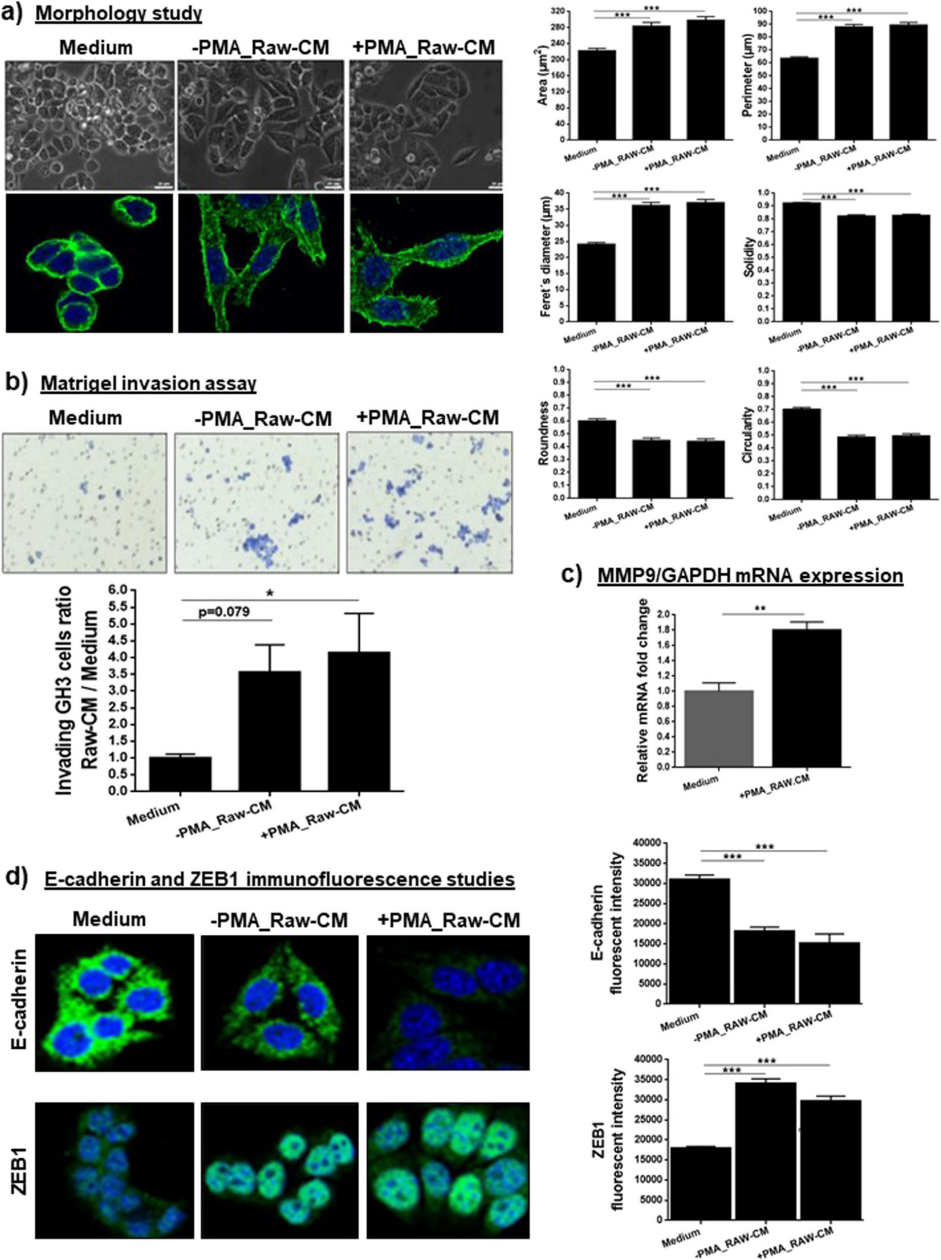
**a** Morphological evaluation of GH3 cells after treatment for 72 h with complete medium and RAW 264.7 macrophage-CM, either from untreated macrophages (−PMA_Raw.CM) or macrophages treated with PMA (+PMA_Raw.CM). Data are shown as mean ± standard error of the mean (SEM) for the 6 morphological parameters evaluated by Image J: cell area (μm^2^), Feret’s diameter (μm), solidity (0–1), perimeter (μm), roundness (0–1) and circularity (0–1). Seventy-five cells were analysed per experiment, with a minimum of 3 experiments per treatment condition. Scale bar 25 μm. ***, <0.001 (one-way ANOVA with Bonferroni multiple comparison test). Alterations on actin cytoskeletal fibers in GH3 cells after treatment with macrophage-CM for 72 h in comparison to complete medium; representative images taken on confocal microscope at 63x magnification; DAPI was used to stain the nuclei. **b** Matrigel-coated chamber invasion assays on GH3 cells towards complete medium, −PMA_Raw.CM and + PMA_Raw.CM after 72 h. Data are shown as mean ± SEM for the ratio of invading GH3 cells towards -PMA_Raw.CM and + PMA_Raw.CM in relation to invading GH3 cells in complete medium conditions. Invasion studies were repeated 4 times in duplicate. *, <0.05 (one-way ANOVA with Bonferroni multiple comparison test). **c** MMP-9 expression in GH3 cells in complete medium or after treatment for 24 h with + PMA_Raw.CM, as determined by RT-qPCR. Data are shown mean ± SEM for the relative MMP9 mRNA fold change expression to GAPDH, as determined by the ∆∆CT method. n = 3. **, <0.01 (Mann Whitney U test). **d** Alterations in E-cadherin and ZEB1 expression by GH3 cells after 72 h treatment with complete medium, −PMA_Raw.CM or + PMA_Raw.CM. Untreated GH3 cells show strong E-cadherin with membranous localisation but also in the cytoplasm as well as low nuclear ZEB1 expression, while macrophage-CM treated GH3 cells display decreased E-cadherin expression and increased nuclear ZEB1 expression. Pictures were taken on confocal microscope at 63x magnification. DAPI was used to stain the nuclei. E-cadherin and ZEB1 fluorescent intensities are shown as mean ± SEM and were quantified in 30 different cells per treatment condition. ***, <0.001 (one-way ANOVA with Bonferroni multiple comparison test)

RAW 264.7 macrophage-CM induced significant changes in GH3 cells: morphology changes, increased invasion, epithelial-to-mesenchymal transition (EMT) activation (Fig. 8) and alteration in cytokine secretion (Additional file 7: Table S4). Macrophage-CM from untreated (−PMA_Raw.CM) or PMA-treated macrophages (+PMA_Raw.CM) increased GH3 cell area, perimeter and Feret’s diameter, and reduced their solidity, roundness and circularity indicating that GH3 cells acquired an EMT-like phenotype. Macrophage-induced morphology changes in GH3 cells were confirmed with immunocytochemistry for actin: GH3 cells treated with macrophage-CM developed a granular pattern of actin with prominent stress fibres and numerous spikes (Fig. 8a), representing an EMT-like cytoskeletal alteration [50].

GH3 cells showed higher invasion towards +PMA_Raw-CM compared to complete medium (Fig. 8b). As invasion depends on cell ability to secrete proteases to degrade extracellular matrix, we hypothesised that macrophage-CM upregulates matrix metalloproteinases (MMPs) expression in GH3 cells allowing them to invade. MMP-9 is a key protease for type IV collagen degradation (main component of Matrigel [32], and of the pituitary capsule and cavernous sinus wall [14, 31, 36]), as well as MMP-9 overexpression is associated with PitNET invasiveness [36]. Hence, we studied MMP-9 expression in GH3 cells after treatment with macrophage-CM, and we observed a significant MMP-9 upregulation in GH3 cells exposed to macrophage-derived factors (Fig. 8c).

Macrophage-CM induced EMT activation in GH3 cells, decreasing E-cadherin and increasing ZEB1 expression (Fig. 8d), two hallmarks of EMT activation [17]. These findings are in line with the morphological changes and invasion assays results, suggesting that macrophage-derived factors interact with pituitary tumour cells influencing their behaviour and invasiveness.

We also found that PMA-activated macrophage-CM induced cytokine secretion changes in GH3 cells, increasing the release of CX3CL1, CCL3, CXCL1, CXCL10, IL-1β, IL-10, IL-13 and VEGF (Additional file 7: Table S4), peptides that play a role in different tumourigenic mechanisms [5, 7, 40, 41].

## Discussion

**Fig. 9:**
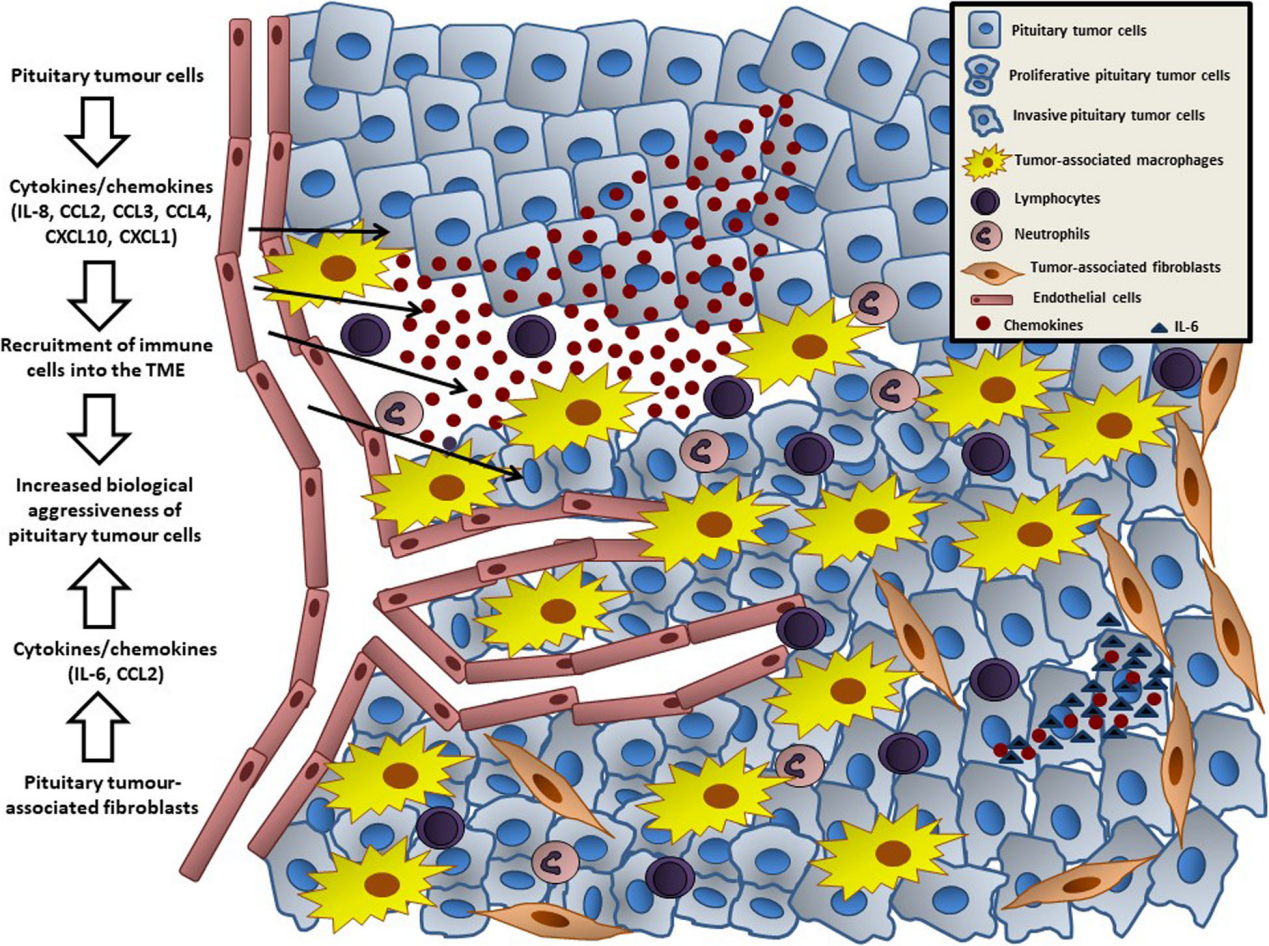
The tumor microenvironment of PitNETs. Pituitary tumour cells release different cytokines, particularly chemokines (including IL-8, CCL2, CCL3, CCL4, CXCL10, CXCL1), directly into the tumour microenvironment promoting the recruitment and infiltration of immune cells, including macrophages, lymphocytes and neutrophils. PitNET-infiltrating immune cells change the behaviour of pituitary tumour cells, namely increasing their proliferative capacity. PitNET-associated fibroblasts are also able to secrete cytokines into the TME, including IL-6 and other chemokines, which in turn lead to increased invasion of pituitary tumour cells

In this study, we found that PitNETs are an active source of cytokines, particularly chemokines, which facilitate macrophage, neutrophil and lymphocyte recruitment into the TME. We found increased macrophage content in the tumours, which together with the other cells provide an inflammatory signature correlating with Ki-67 staining. Our in vitro cell model data confirmed the macrophage chemoattractant effect of pituitary tumour-derived factors, while macrophage-derived factors influence tumour cell behaviour leading to morphological changes, increased invasion, cytokine secretome changes and EMT activation. Thus, the cytokine network within the TME of PitNETs, derived from both tumour and immune cells, as well as from PitNET-associated fibroblasts [47], may play a role in the modulation of the TME and in the aggressiveness of PitNETs (Fig. 9).

IL-8, CCL2, CCL3 and CCL4 were highly secreted by the majority of PitNETs. Cytokine array data from human craniopharyngiomas identified CCL2 and IL-8 as the most secreted cytokines in plasma, primary culture supernatants, cell and tissue lysates [57]. There are no previous data on CCL2, CCL3 or CCL4 in PitNETs, but these chemokines are involved in tumour growth and invasion in other tumours [5, 41], as well as in immune cell chemotaxis in cancer [5, 12, 16, 41, 43, 63, 72, 92]. We found no association between CCL2, CCL3 or CCL4 levels and PitNET aggressiveness, but PitNETs with a higher content of macrophages, CD8+ T cells and neutrophils secreted higher chemokine levels. PitNETs with more macrophages were associated with higher levels of IL-8, a chemokine that influences several oncogenic pathways [1, 86]. IL-8 mRNA was previously found in PitNETs, although different detection methods provided a wide range of expression levels [23, 76, 84]. We also identified other chemokines potentially relevant in PitNETs, namely CXCL10, CCL22, CXCL1 and CX3CL1. Our data suggest CXCL1 and CXCL10 as potential modulators of PitNET-infiltrating neutrophils and macrophages. CXCL1 and its receptor CXCR2 were previously identified in human PitNETs [79], but there are no data regarding CXCL10, CCL22 and CX3CL1. Together, these findings suggest a link between endocrine cells and chemokines reflecting their possible involvement in tumourigenesis and modulation of immune infiltrates, as well as a promising target for drugs affecting the PitNET cytokine network, as already explored for other cancers [7, 41, 67].

Macrophages are present in normal [29, 39] and neoplastic pituitary [10, 21, 37]. Our immunohistochemical and xCELL data showed that PitNETs contained 3-4x more macrophages than NPs, and are the predominant immune cell type in PitNETs. We found no association between PitNET-infiltrating macrophages and cavernous sinus invasion, and the correlation with high Ki-67 was borderline. Lu et al. reported that macrophage content was correlated with size and invasiveness [37]. *AIP* mutation-positive somatotropinomas, often more aggressive [45, 46], have more PitNETs-infiltrating macrophages than sporadic somatotropinomas or NPs [10].

Next, we studied the phenotype of infiltrating macrophages in human PitNETs and NPs using CD163 (M2-like) and HLA-DR (M1-like) macrophage markers [44, 71, 81]. We noted a 3-fold increased CD163:HLA-DR ratio in PitNETs compared to NPs, in line with our xCell data (score for M2-macrophages was >4x higher in PitNETs). The predominance of M2-macrophages in PitNETs can be due, at least in part, to higher concentrations of PitNET-derived M2-polarising cytokines, namely IL-4, which was ~5x higher than IFNγ, the main M1-polarising cytokine [42, 44]. M1- and M2-like macrophages have been described in normal rat pituitary and in diethylstilbestrol-induced prolactinomas [21], with prolactinomas having remarkably more M2-macrophages than NP. M2-macrophages number increased during the first weeks of diethylstilbestrol treatment, even before tumour formation, suggesting a role for M2-macrophages in initiating tumourigenesis. During diethylstilbestrol treatment capillaries became more tortuous with increased calibre and developed haemorrhage areas, suggesting a possible role for M2-macrophages in angiogenesis and vasculature modulation in PitNETs [21], in agreement with our observed correlations between CD163:HLA-DR macrophage ratio and PitNET microvessel density and area. These findings support a role for infiltrating M2-macrophages in the angiogenesis of PitNETs, as previously described in other cancers [13, 27, 42, 44, 80, 83]. We found no association between PitNET-infiltrating CD163+ macrophages and cavernous sinus invasion, although a recent study showed more CD163+ macrophages in invasive NF-PitNETs than in non-invasive tumours [68].

We found that a low CD8:CD4 ratio is associated with higher Ki-67, suggesting that relatively low CD8+ to high CD4+ T cells, rather than absolute CD8+ and CD4+ T cell amounts per se, represent a relative imbalance potentially affecting tumour proliferation. This has been previously described in gliomas, where the number of tumour-infiltrating CD8+ and CD4+ cells alone had no prognostic value, while the presence of a low CD8:CD4 ratio was an independent predictor for poor progression-free and overall survival [26]. Poor clinical outcome and persistence/recurrence was described in PitNETs with tumour-infiltrating lymphocytes [38]. Another study found no association between CD8+ T cell count and Ki-67, tumour size, gender or age [85]. We observed more CD4+ and fewer CD8+ cells, with a significant 2-fold decrease in CD8:CD4 ratio in comparison to NPs, supporting the known anti-tumoural role of cytotoxic CD8+ T cells and the pro-tumoural CD4+ T cells, possibly Th2 [9, 25, 26]. Indeed, downregulation of Th1 pathway-related genes was observed in aggressive PitNETs [64]. CD163+ macrophages, the predominant form we observed in PitNETs, support CD4+ Th2 cells and prevent the expression of cytokines required for CD4+ Th1 cells [37, 54], which may further contribute to a PitNET-associated Th2 phenotype. Visa versa, CD4+ Th2 cytokines in the TME sustain M2 macrophages [43, 44, 54] possibly contributing to the CD163+ macrophage phenotype we observed in PitNETs.

Although we found generally low amounts of FOXP3+ T cells in PitNETs, as previously shown [30], PitNETs with higher Ki-67 had significantly more FOXP3+ T cells. Moreover, a significant 3-fold reduced CD8:FOXP3 ratio was noted in PitNETs with a higher Ki-67, revealing that a deleterious imbalance between CD8+ and FOXP3+ T cells may lead to increased proliferation and thereby aggressiveness, as described for other cancers [70, 77]. In our study, all PitNETs with a “deleterious immune phenotype”, i.e. higher content of macrophages, T helper lymphocytes, FOXP3+ T regulatory and B cells, had a Ki-67 ≥ 3%, which together with results regarding cell ratios CD68:FOXP3, CD8:CD4 and CD8:FOXP3, highlights that the pooled inflammatory context integrating different immune subpopulations within the TME of PitNETs is more relevant for biological behaviour and aggressiveness than each PitNET-infiltrating immune cell per se.

We found significantly less neutrophils in PitNETs than NPs. Somatotropinomas had fewer neutrophils than NF-PitNETs which could be due to less chemokine release, particularly IL-8, as suggested by our data, but other factors can be also involved such as impaired neutrophil chemotaxis in acromegaly [20].

There is some variability in the PitNETs immune infiltrates reported in the literature [28, 37, 38, 52, 65, 85]. This variability can reflect the variable level of immunosurveillance from tumour to tumour [9, 74, 75], patient selection [37], or can be due to lack of standardisation method reporting immune infiltrates [37, 38, 85], such as reporting hot spots or taking random HPFs, or reporting interstitial areas or perivascular inflammatory cells [37], usage of different cell markers and antibodies to detect the same immune cell type [28, 37, 38], or assessment of full slides versus tissue microarrays, which can all greatly influence the results. Despite these issues, our immunohistochemical data are in agreement with our xCELL data, and are generally in line with the previously published data [37, 38, 52, 85].

Our in vitro cell line experiments focused on macrophages as these are the predominant immune cell type in PitNETs, and we selected RAW 264.7 macrophages for a number of reasons: (i) lack of a reliable rat macrophage cell line; (ii) high homology between mouse and rat cytokines; (iii) expression of CX3CR1 [22], the chemokine receptor for CX3CL1 which was the main GH3 cell-derived chemokine according to our cytokine array data and (iv) to validate some of our previous observations on a different cell model employing primary bone-marrow derived rat macrophages and GH3 cells [10]. Our in vitro observations here reported, consistent with our previous findings on a different cell model [10], show a remarkable macrophage chemoattractant effect induced by GH3 cell-derived factors, an effect explained not only by the chemokine gradient but also by their ability to upregulate chemokine receptor expression. These findings are in line with our human data (association between PitNET-infiltrating macrophages and higher PitNET-derived chemokine levels), suggesting that PitNET cells could attract macrophages. In turn, we showed that macrophage-derived factors induced numerous effects on GH3 cells, including changes in morphology, invasion, EMT activation and cytokine secretome alterations, suggesting that immune cell-derived factors influence tumour mechanisms and lead to increased PitNET aggressiveness, as shown in other cancers [5, 7, 40, 41].

Our study, using a comprehensively phenotyped cohort of human samples with cytokine array data from primary culture, immunohistochemical immune cell infiltrates and clinicopathological data, found an association between PitNET-derived chemokines and infiltrating immune cells, particularly macrophages, CD8+ T cells and neutrophils. These data suggest a potential role for immune infiltrates to determine PitNET aggressiveness, particularly proliferation. Our human data are strengthened by our in vitro functional data providing mechanistic insights into the crosstalk between pituitary tumour cells and macrophages.

Limitations of our study include the fact that we have a relatively small cohort of cases, and thus our observations need to be validated in larger series preferably including all different PitNET types. As the study is based on fresh primary culture, we inevitably have a relatively short postoperative follow-up of the patients, rendering data on recurrence unavailable. In our in vitro data we used a rat rather than human cell line, as a human pituitary cell line does not exist; moreover, our monolayer cell cultures are unable to investigate the complex paracrine and autocrine interactions occurring in vivo within the TME, which involves a wider range of immune cells besides macrophages, as well as stromal cells, endothelial cells, pericytes and the extracellular matrix [6]. We followed a CD163+ and HLA-DR+ distinction between macrophages [27, 42, 44], although we acknowledge that is simplistic and may not comprehensively address the heterogeneous and complex macrophage phenotypes representing the wide spectrum of macrophages [55]. There is a considerable heterogeneity on methodology used to study tumour-associated macrophages, particularly regarding their surface markers. We selected CD68 which satisfactorily identify general macrophages, and CD163 to identify alternatively activated macrophages [27, 42, 44]. Studying classically activated macrophages is more challenging, as a specific marker is lacking, but HLA-DR or iNOS (inducible nitric oxide synthase) are often used for this purpose [34, 44, 51]. Thus, our findings from the immunohistochemical study may well be partially influenced by such elements; nevertheless, our data were reproduced on a separate set of samples using a different methodology (the gene-signature based xCell), providing another layer of evidence regarding the macrophage phenotype of human PitNETs.

## Conclusions

Our data suggest that pituitary tumour cells release cytokines, particularly chemokines, which facilitate immune cell recruitment into the tumour microenvironment of PitNETs. The increased inflammatory signature could influence tumour cell proliferation. We found increased macrophage migration towards pituitary tumour cell-derived factors in vitro, and in turn, macrophage secreting-factors change pituitary cells phenotype inducing epithelial-to-mesenchymal transition, increased invasion and altered cytokine secretion. Our study provide novel insights into the PitNET biology, and provide novel targets for treatment of aggressive PitNETs.

## Supplementary information

**Additional file 1:** **Figure S1.** a) Cytokine secretome from somatotropinomas treated pre-operatively with somatostatin analogues (Pre-op SSA, *n* = 5) vs not treated (No pre-op SSA, *n* = 3). b) Cytokine secretome from densely granulated (*n* = 3) vs sparsely granulated (*n* = 5) somatotropinomas. Data are shown for the top 12 secreted proteins as mean concentration ± standard error of the mean. No significant differences were found (Mann Whitney U test).

**Additional file 2:** **Figure S2.** xCELL Fraction Scores obtained from microarray expression data from a different set of samples (7 PitNETs - 4 NF-PitNETs and 3 somatotropinomas - and 5 NPs). Data are shown in mean xCELL Fraction Score ± standard error of the mean. Comparative analysis was carried out for the immune cell types originally analysed by immunohistochemistry in our cohort. *, < 0.05, **, < 0.01, ***, < 0.001 (Mann Whitney U test).

**Additional file 3:** **Figure S3.** Correlation between PitNET tissue immune cell infiltrates and the respective circulating immune cell subpopulation. *n* = 24. *P* values were determined by the Pearson correlation.

**Additional file 4:** **Table S1.** Primary antibodies and respective dilutions used for immunohistochemical (IHC) and immunofluorescence (IF) studies.

**Additional file 5:** **Table S2.** Cytokine secretome from the 24 human PitNETs-derived cell culture supernatants. PitNETs-derived supernatants were collected at 24 h on serum-free medium conditions and cytokine secretome determined with the human Millipore MILLIPLEX cytokine 42-plex array. Data are shown as mean concentration (pg/mL) ± standard error of the mean (SEM) for all detectable cytokines/ chemokines/ growth factors. IL-1ra, IL-2, IL-3, IL-5, IL-7, IL-9, IL-13, CCL7, sCD40L, TNF-β and TGF-α were undetectable in PitNETs-derived supernatants.

**Additional file 6:** **Table S3.** Immunohistochemical analysis of the immune cells and respective ratios among the various NF-PitNET types, and subgroup comparative analysis between NF-PitNETs vs somatotropinomas. Immune cells analysed: macrophages (CD68+), CD163+ macrophages, HLA-DR macrophages, cytotoxic T lymphocytes (CD8+), T helper lymphocytes (CD4+), T regulatory cells (FOXP3+), B cells (CD20+) and neutrophils (neutrophil elastase+). Data are shown as mean ± standard error of the mean for percentage of immune cells compared to the total number of tumour cells and for cell ratios. One way-ANOVA test was used to calculate *p* value among the NF-PitNETs histiotypes: gonadotroph PitNET, silent corticotroph PitNET and null cell PitNET (GP vs SCP vs NCP). Mann Whitney U test was used to calculate *p* value for the comparison NF-PitNETs vs somatotropinomas (NF vs Som).

**Additional file 7:** **Table S4.** Cytokine secretome from GH3 cells at baseline (untreated) and after treatment with PMA-activated RAW 264.7 macrophage-CM (+PMA_Raw-CM) for 24 h (*n* = 3). Data are shown in concentration (pg/mL) ± standard error of the mean (SEM) for the cytokines/chemokines/growth factors with detectable concentrations as identified by the rat Millipore MILLIPLEX cytokine 27-plex array. CCL5, CCL11, G-CSF, GM-CSF, IL-1α, IL-12, IL-17A, TNF-α, EGF, Leptin and LIX were not detected in the GH3 supernatants (i.e. concentration below the lowest standard curve point and/or serum-free medium quantification). Mean ratio ± SEM between untreated vs + PMA_Raw-CM GH3 cells-treated is also shown in the table, with significant *p* values indicated in the same column as asterisks. *,< 0.05, **,< 0.01 (Mann Whitney U test).

## Data Availability

Microarray data used for xCell analysis have been deposited with the National Center for Biotechnology Information Gene Expression Omnibus (www.ncbi.nlm.nih.gov/geo, accession number GSE63357).

## Abbreviations

CM: Conditioned medium
CT: Cycle threshold
DMEM: Dulbecco’s Modified Eagle’s Medium
EMT: Epithelial-to-mesenchymal transition
FBS: Foetal bovine serum
HPF: High power field
iNOS: Inducible nitric oxide synthase
MMP: Matrix metalloproteinase
NF-PitNET: Non-functioning pituitary neuroendocrine tumour
NP: Normal pituitary
PBS: Phosphate Buffered Saline
PitNET: Pituitary neuroendocrine tumour
PMA: Phorbol 12-Myristate 13-Acetate
RT-qPCR: Real-time quantitative polymerase chain reaction
TME: Tumour microenvironment
