# Supplementary figures and images for: Chemokines modulate the tumour microenvironment in pituitary neuroendocrine tumours
Source: Acta Neuropathol Commun. 2019 Nov 8;7:172. doi: 10.1186/s40478-019-0830-3 (PMC6839241; doi:10.1186/s40478-019-0830-3)

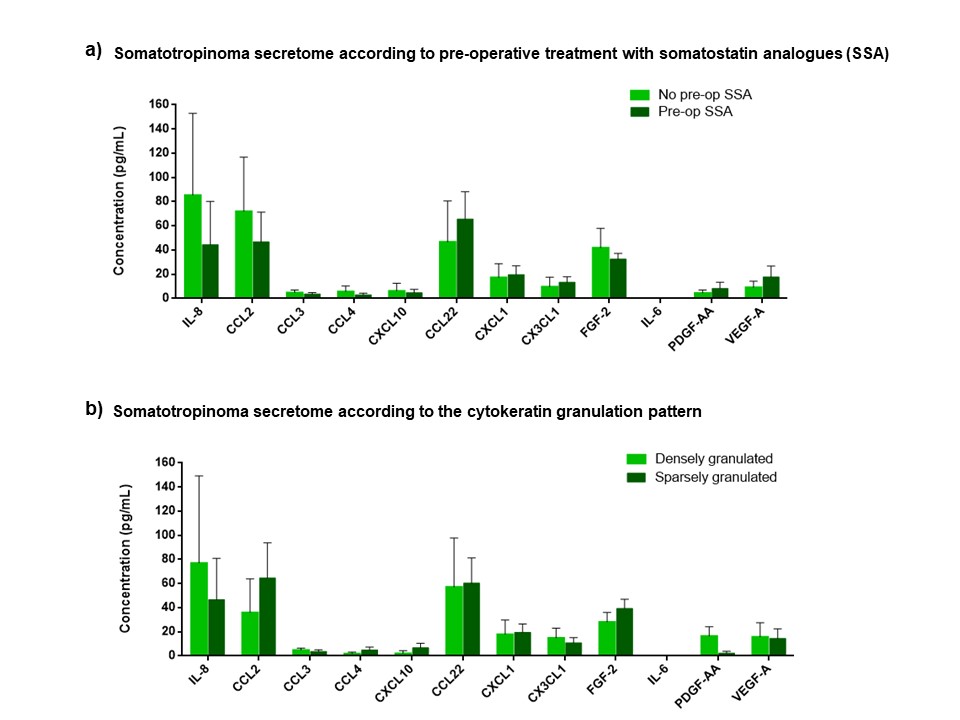

Supplement: Supplementary file 1 — Additional file 1: Figure S1. a) Cytokine secretome from somatotropinomas treated pre-operatively with somatostatin analogues (Pre-op SSA, n = 5) vs not treated (No pre-op SSA, n = 3). b) Cytokine secretome from densely granulated (n = 3) vs sparsely granulated (n = 5) somatotropinomas. Data are shown for the top 12 secreted proteins as mean concentration ± standard error of the mean. No significant differences were found (Mann Whitney U test). [file 40478_2019_830_MOESM1_ESM.jpg]

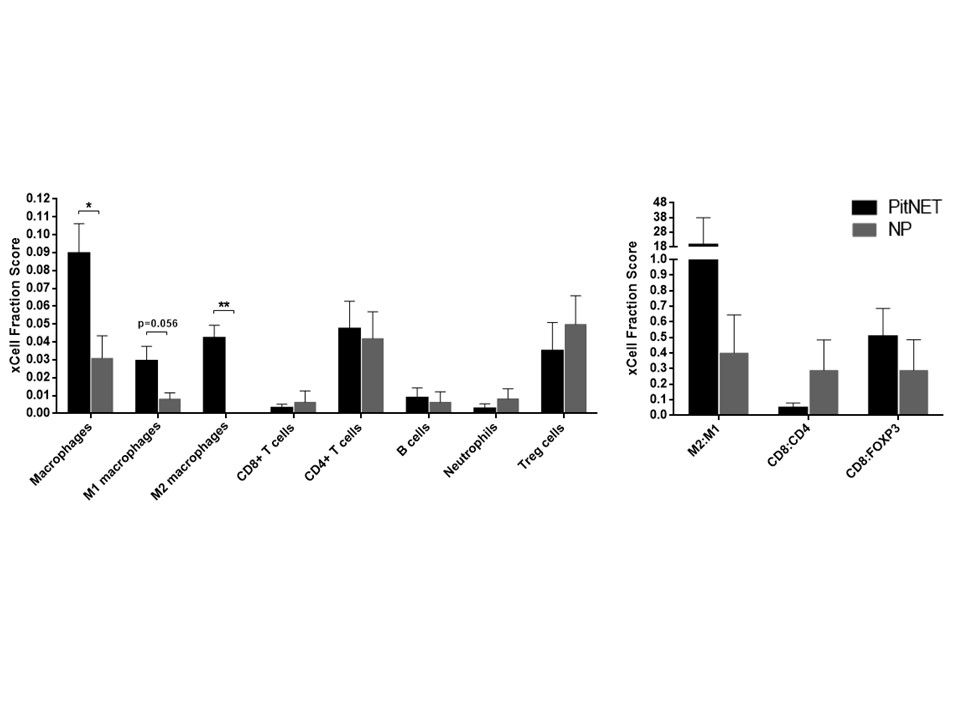

Supplement: Supplementary file 2 — Additional file 2: Figure S2. xCELL Fraction Scores obtained from microarray expression data from a different set of samples (7 PitNETs - 4 NF-PitNETs and 3 somatotropinomas - and 5 NPs). Data are shown in mean xCELL Fraction Score ± standard error of the mean. Comparative analysis was carried out for the immune cell types originally analysed by immunohistochemistry in our cohort. *, < 0.05, **, < 0.01, ***, < 0.001 (Mann Whitney U test). [file 40478_2019_830_MOESM2_ESM.jpg]

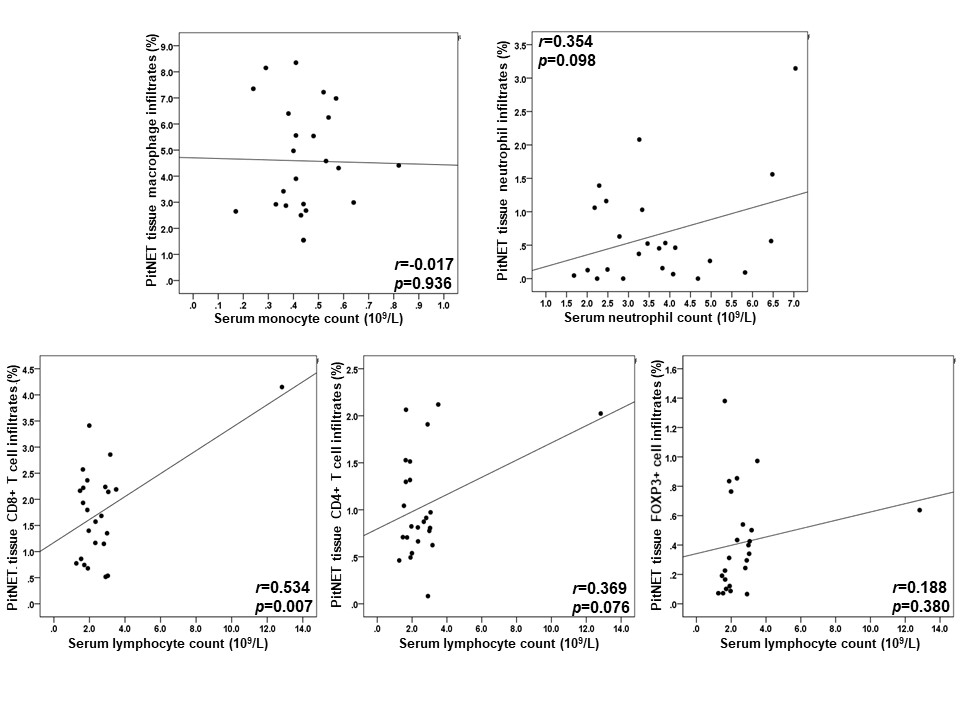

Supplement: Supplementary file 3 — Additional file 3: Figure S3. Correlation between PitNET tissue immune cell infiltrates and the respective circulating immune cell subpopulation. n = 24. P values were determined by the Pearson correlation. [file 40478_2019_830_MOESM3_ESM.jpg]
