# Supplementary material for: Chemokines modulate the tumour microenvironment in pituitary neuroendocrine tumours
Source: Acta Neuropathol Commun. 2019 Nov 8;7:172. doi: 10.1186/s40478-019-0830-3 (PMC6839241; doi:10.1186/s40478-019-0830-3)
Supplement: Supplementary file 4 — Additional file 4: Table S1. Primary antibodies and respective dilutions used for immunohistochemical (IHC) and immunofluorescence (IF) studies. [file 40478_2019_830_MOESM4_ESM.docx]

| **Antibody** | **Company** | **Cat. no.** | **Species** | **Dilution IHC** | **Dilution IF** |
| --- | --- | --- | --- | --- | --- |
| **Actin** | Molecular Probes | R37110 | Mouse |  | 2 drops/mL (1:500) |
| **CD4** | Abcam | Ab133616 | Rabbit | 1:100 |  |
| **CD8** | DAKO | M7103 | Mouse | 1:100 |  |
| **CD20** | DAKO | M0755 | Mouse | 1:300 |  |
| **CD31** | DAKO | M0823 | Mouse | 1:100 |  |
| **CD68** | DAKO | IR613 | Mouse | 1:2 |  |
| **CD163** | Abcam | Ab74604 | Mouse | Neat |  |
| **E-cadherin** | BD Biosciences | 610181 | Mouse |  | 1:50 |
| **FOXP3** | Abcam | Ab20034 [236A/E7] | Mouse | 1:50 |  |
| **HLA-DR** | Abcam | Ab20181 [TAL1B5] | Mouse | 1:100 |  |
| **Neutrophil elastase** | Abcam | Ab68672 | Rabbit | 1:100 |  |
| **ZEB1** | Santa Cruz Biotechnology | H-102: sc-25388 | Rabbit |  | 1:50 |

**Additional file 4: Table S1:** **Primary antibodies and respective dilutions used for immunohistochemical (IHC) and immunofluorescence (IF) studies.**
