# Supplementary material for: Chemokines modulate the tumour microenvironment in pituitary neuroendocrine tumours
Source: Acta Neuropathol Commun. 2019 Nov 8;7:172. doi: 10.1186/s40478-019-0830-3 (PMC6839241; doi:10.1186/s40478-019-0830-3)
Supplement: Supplementary file 5 — Additional file 5: Table S2. Cytokine secretome from the 24 human PitNETs-derived cell culture supernatants. PitNETs-derived supernatants were collected at 24 h on serum-free medium conditions and cytokine secretome determined with the human Millipore MILLIPLEX cytokine 42-plex array. Data are shown as mean concentration (pg/mL) ± standard error of the mean (SEM) for all detectable cytokines/ chemokines/ growth factors. IL-1ra, IL-2, IL-3, IL-5, IL-7, IL-9, IL-13, CCL7, sCD40L, TNF-β and TGF-α were undetectable in PitNETs-derived supernatants. [file 40478_2019_830_MOESM5_ESM.docx]

| **Cytokine/ Chemokine/ Growth factor** | **Mean concentration (pg/mL) ± SEM** | **Serum-free medium (pg/mL)** |
| --- | --- | --- |
| **IL-8** | 854.18 ± 445.79 | 7.06 |
| **CCL2** | 578.03 ± 222.66 | 4.00 |
| **CCL3** | 150.55 ± 88.22 | 0 |
| **CCL4** | 94.25 ± 47.20 | 3.09 |
| **CXCL10** | 76.67 ± 47.58 | 0 |
| **CCL22** | 67.25 ± 16.74 | 20.78 |
| **CXCL1** | 60.30 ± 26.14 | 20.78 |
| **CX3CL1** | 35.14 ± 17.12 | 6.73 |
| **FGF-2** | 26.65 ± 4.11 | 0 |
| **IL-6** | 24.90 ± 19.27 | 0 |
| **PDGF-AA** | 22.36 ± 6.78 | 0.12 |
| **VEGF-A** | 15.85 ± 4.06 | 0 |
| **PDGF-BB** | 13.37 ± 6.09 | 0 |
| **IFNα2** | 4.90 ± 1.00 | 1.79 |
| **IL-4** | 4.75 ± 1.47 | 0 |
| **G-CSF** | 3.97 ± 1.25 | 0 |
| **GM-CSF** | 3.89 ± 1.71 | 0 |
| **CCL5** | 3.83 ± 0.97 | 0.66 |
| **IL-12p40** | 3.66 ± 0.96 | 0 |
| **TNF-α** | 3.01 ± 2.24 | 0.19 |
| **IL-18** | 2.87 ± 0.66 | 1.91 |
| **Flt3L** | 2.79 ± 0.34 | 1.72 |
| **CCL11** | 2.77 ± 0.66 | 0 |
| **IL-1α** | 1.75 ± 0.70 | 0 |
| **EGF** | 1.69 ± 0.43 | 0 |
| **IFNγ** | 1.23 ± 0.24 | 0.34 |
| **IL-10** | 1.16 ± 0.39 | 0.55 |
| **IL-1β** | 0.90 ± 0.24 | 0.06 |
| **IL-12p70** | 0.86 ± 0.22 | 0.11 |
| **IL-15** | 0.76 ± 0.20 | 0.55 |
| **IL-17A** | 0.68 ± 0.12 | 0.01 |

**Additional file 5: Table S2:** **Cytokine secretome from the 24 human PitNETs-derived cell culture supernatants.** PitNETs-derived supernatants were collected at 24h on serum-free medium conditions and cytokine secretome determined with the human Millipore MILLIPLEX cytokine 42-plex array. Data are shown as mean concentration (pg/mL) ± standard error of the mean (SEM) for all detectable cytokines/ chemokines/ growth factors. IL-1ra, IL-2, IL-3, IL-5, IL-7, IL-9, IL-13, CCL7, sCD40L, TNF-β and TGF-α were undetectable in PitNETs-derived supernatants.
