# Supplementary material for: Chemokines modulate the tumour microenvironment in pituitary neuroendocrine tumours
Source: Acta Neuropathol Commun. 2019 Nov 8;7:172. doi: 10.1186/s40478-019-0830-3 (PMC6839241; doi:10.1186/s40478-019-0830-3)
Supplement: Supplementary file 6 — Additional file 6: Table S3. Immunohistochemical analysis of the immune cells and respective ratios among the various NF-PitNET types, and subgroup comparative analysis between NF-PitNETs vs somatotropinomas. Immune cells analysed: macrophages (CD68+), CD163+ macrophages, HLA-DR macrophages, cytotoxic T lymphocytes (CD8+), T helper lymphocytes (CD4+), T regulatory cells (FOXP3+), B cells (CD20+) and neutrophils (neutrophil elastase+). Data are shown as mean ± standard error of the mean for percentage of immune cells compared to the total number of tumour cells and for cell ratios. One way-ANOVA test was used to calculate p value among the NF-PitNETs histiotypes: gonadotroph PitNET, silent corticotroph PitNET and null cell PitNET (GP vs SCP vs NCP). Mann Whitney U test was used to calculate p value for the comparison NF-PitNETs vs somatotropinomas (NF vs Som). [file 40478_2019_830_MOESM6_ESM.docx]

|  | Gonadotroph PitNET (n=13) | Silent Corticotroph PitNET (n=1) | Null Cell PitNET (n=2) | *p* value  (GP vs SCP vs NCP) | NF-PitNETs (n=16) | Somatotropinomas (n=8) | *p* value  (NF vs Som) |
| --- | --- | --- | --- | --- | --- | --- | --- |
| % of macrophages | 4.23 ± 0.60 | 4.97 | 6.37 ± 1.79 | 0.456 | 4.54 ± 0.54 | 4.66 ± 0.70 | 0.897 |
| % of CD8+ T cells | 1.62 ± 0.29 | 1.17 | 1.76 ± 0.61 | 0.891 | 1.61 ± 0.24 | 2.09 ± 0.29 | 0.245 |
| % of CD4+ T cells | 1.10 ± 0.17 | 0.81 | 1.12 ± 0.20 | 0.897 | 1.09 ± 0.14 | 0.96 ± 0.20 | 0.629 |
| % of B cells | 1.03 ± 0.48 | 0.66 | 0.68 ± 0.18 | 0.946 | 0.97 ± 0.39 | 0.84 ± 0.33 | 0.836 |
| % of neutrophils | 1.05 ± 0.24 | 0.09 | 0.50 ± 0.13 | 0.420 | 0.92 ± 0.21 | 0.14 ± 0.06 | **0.002** |
| % of FOXP3+ T cells | 0.34 ± 0.10 | 0.85 | 0.28 ± 0.34 | 0.372 | 0.37 ± 0.09 | 0.52 ± 0.11 | 0.310 |
| CD163:HLA-DR ratio | 2.36 ± 0.22 | 1.98 | 1.96 ± 0.77 | 0.752 | 2.29 ± 0.19 | 1.98 ± 0.25 | 0.351 |
| CD8:CD4 ratio | 1.79 ± 0.38 | 1.33 | 1.50 ± 0.23 | 0.391 | 1.73 ± 0.31 | 2.62 ± 0.60 | 0.154 |
| CD8: FOXP3 ratio | 6.12 ± 1.12 | 1.33 | 5.08 ± 0.42 | 0.678 | 5.69 ± 0.95 | 5.56 ± 1.25 | 0.932 |

**Additional file 6: Table S3: Immunohistochemical analysis of the immune cells and respective ratios among the various NF-PitNET types, and subgroup comparative analysis between NF-PitNETs vs somatotropinomas.** Immune cells analysed: macrophages (CD68+), CD163+ macrophages, HLA-DR macrophages, cytotoxic T lymphocytes (CD8+), T helper lymphocytes (CD4+), T regulatory cells (FOXP3+), B cells (CD20+) and neutrophils (neutrophil elastase+). Data are shown as mean ± standard error of the mean for percentage of immune cells compared to the total number of tumour cells and for cell ratios. One way-ANOVA test was used to calculate *p* value among the NF-PitNETs histiotypes: gonadotroph PitNET, silent corticotroph PitNET and null cell PitNET (GP vs SCP vs NCP). Mann Whitney U test was used to calculate *p* value for the comparison NF-PitNETs vs somatotropinomas (NF vs Som).
