# Supplementary material for: Chemokines modulate the tumour microenvironment in pituitary neuroendocrine tumours
Source: Acta Neuropathol Commun. 2019 Nov 8;7:172. doi: 10.1186/s40478-019-0830-3 (PMC6839241; doi:10.1186/s40478-019-0830-3)
Supplement: Supplementary file 7 — Additional file 7: Table S4. Cytokine secretome from GH3 cells at baseline (untreated) and after treatment with PMA-activated RAW 264.7 macrophage-CM (+PMA_Raw-CM) for 24 h (n = 3). Data are shown in concentration (pg/mL) ± standard error of the mean (SEM) for the cytokines/chemokines/growth factors with detectable concentrations as identified by the rat Millipore MILLIPLEX cytokine 27-plex array. CCL5, CCL11, G-CSF, GM-CSF, IL-1α, IL-12, IL-17A, TNF-α, EGF, Leptin and LIX were not detected in the GH3 supernatants (i.e. concentration below the lowest standard curve point and/or serum-free medium quantification). Mean ratio ± SEM between untreated vs + PMA_Raw-CM GH3 cells-treated is also shown in the table, with significant p values indicated in the same column as asterisks. *,< 0.05, **,< 0.01 (Mann Whitney U test). [file 40478_2019_830_MOESM7_ESM.docx]

| **Cytokine / Chemokine / Growth factor** | **GH3 cells**  **Untreated**  Mean concentration  (pg/mL) ± SEM | **GH3 cells**  **+PMA_Raw-CM**  Mean concentration  (pg/mL) ± SEM | **Ratio GH3 cells**  **+PMA_Raw-CM : Untreated**  Mean ratio ± SEM |
| --- | --- | --- | --- |
| **CX3CL1** | 301.80 ± 6.76 | 400.33 ± 31.39 | 1.33 ± 0.10 * |
| **CCL2** | 81.61 ± 25.47 | 48.29 ± 25.04 | 0.59 ± 0.31 |
| **CCL3** | 0.92 ± 0.80 | 6.46 ± 0.89 | 7.04 ± 0.97 ** |
| **CXCL1** | 16.17 ± 4.64 | 135.86 ± 12.31 | 8.40 ± 0.76 ** |
| **CXCL2** | 29.67 ± 2.33 | 7.77 ± 7.77 | 0.26 ± 0.26 * |
| **CXCL10** | 6.65 ± 0.55 | 24.26 ± 1.40 | 3.65 ± 0.21 ** |
| **IL-1β** | 6.59 ± 1.01 | 17.32 ± 2.22 | 2.63 ± 0.34 * |
| **IL-2** | 20.57 ± 2.33 | 15.07 ± 9.19 | 0.73 ± 0.45 |
| **IL-4** | 6.72 ± 0.90 | 6.95 ± 1.43 | 1.03 ± 0.21 |
| **IL-5** | 11.77 ± 1.09 | 6.72 ± 4.46 | 0.57 ± 0.38 |
| **IL-6** | 168.97 ± 31.48 | 169.42 ± 98.92 | 1.00 ± 0.59 |
| **IL-10** | 6.71 ± 2.65 | 262.96 ± 35.55 | 39.81 ± 5.38 ** |
| **IL-13** | 6.30 ± 1.06 | 11.83 ± 2.99 | 1.88 ± 0.47 * |
| **IL-18** | 3.96 ± 1.93 | 10.68 ± 5.96 | 2.70 ± 1.50 |
| **VEGF** | 2534.20 ± 97.52 | 4521.34 ± 548.86 | 1.78 ± 0.22 * |
| **IFNγ** | 589.94 ± 118.03 | 494.26 ± 42.90 | 0.62 ± 0.09 |

**Additional file 7: Table S4: Cytokine secretome from GH3 cells at baseline (untreated) and after treatment with PMA-activated RAW 264.7 macrophage-CM (+PMA_Raw-CM) for 24h (n=3).** Data are shown in concentration (pg/mL) ± standard error of the mean (SEM) for the cytokines/chemokines/growth factors with detectable concentrations as identified by the rat Millipore MILLIPLEX cytokine 27-plex array. CCL5, CCL11, G-CSF, GM-CSF, IL-1α, IL-12, IL-17A, TNF-α, EGF, Leptin and LIX were not detected in the GH3 supernatants (i.e. concentration below the lowest standard curve point and/or serum-free medium quantification). Mean ratio ± SEM between untreated vs +PMA_Raw-CM GH3 cells-treated is also shown in the table, with significant *p* values indicated in the same column as asterisks. *,<0.05, **,<0.01 (Mann Whitney U test).
